# Combined PD-L1 and TIM3 blockade improves expansion of fit human CD8^+^ antigen-specific T cells for adoptive immunotherapy

**DOI:** 10.1016/j.omtm.2022.09.016

**Published:** 2022-10-04

**Authors:** Shirin Lak, Valérie Janelle, Anissa Djedid, Gabrielle Boudreau, Ann Brasey, Véronique Lisi, Ali Smaani, Cédric Carli, Lambert Busque, Vincent-Philippe Lavallée, Jean-Sébastien Delisle

**Affiliations:** 1 Centre de Recherche de L’Hôpital Maisonneuve-Rosemont, 5415 Boul. de L’Assomption, Montréal, QC H1T 2M4, Canada; 2 Centre de Recherche Du CHU Sainte-Justine, 3175 Chemin de la Côte-Sainte-Catherine, Montréal, QC H3T 1C5, Canada; 3 Biomarker Unit, Centre C3i, 5415 Boul. de L’Assomption, Montréal, QC H1T 2M4, Canada; 4 Department of Medicine, Université de Montréal, CP 6128, Succursale Centre-ville, Montréal, QC H3C 3J7, Canada; 5 Hematology-Oncology and Cell Therapy Division, Hôpital Maisonneuve-Rosemont, Montréal, QC Canada; 6 Department of Pediatrics, Université de Montréal, Montréal, QC, Canada; 7 Hematology-Oncology Division, CHU Sainte-Justine, 3175 Chemin de la Côte-Sainte-Catherine, Montréal, QC H3T 1C5, Canada

**Keywords:** PD-1, TIM3, adoptive immunotherapy, immune checkpoint, T cell manufacturing, single-cell RNA sequencing, T cell exhaustion, WT1, Epstein-Barr virus, antigen-specific T cells

## Abstract

Antigen-specific T cell expansion *ex vivo* followed by adoptive transfer enables targeting of a multitude of microbial and cancer antigens. However, clinical-scale T cell expansion from rare precursors requires repeated stimulation, which may lead to T cell dysfunction and limited therapeutic potential. We used a clinically compliant protocol to expand Epstein-Barr virus (EBV) and Wilms tumor 1 (WT1) antigen-specific CD8^+^ T cells, and leveraged T cell exhaustion-associated inhibitory receptor blockade to improve T cell expansion. Several inhibitory receptors were expressed early by *ex vivo*-expanded antigen-specific CD8^+^ T cells, including PD-1 and TIM3, with co-expression matching evidence of T cell dysfunction as the cultures progressed. Introduction of anti-PD-L1 and anti-TIM3 blockade in combination (but not individually) to the culture led to markedly improved antigen-specific T cell expansion without inducing T cell dysfunction. Single-cell RNA sequencing (RNA-seq) and T cell receptor (TCR) repertoire profiling revealed that double blockade does not impart specific transcriptional programs in T cells or alterations in TCR repertoires. However, combined blockade may affect gene expression in a minority of clonotypes in a donor-specific fashion. We conclude that antigen-specific CD8^+^ T cell manufacturing can be improved by using TIM3 and PD-L1/PD-1 axis blockade in combination. This approach is readily applicable to several adoptive immunotherapy strategies.

## Graphical abstract

**Figure:**
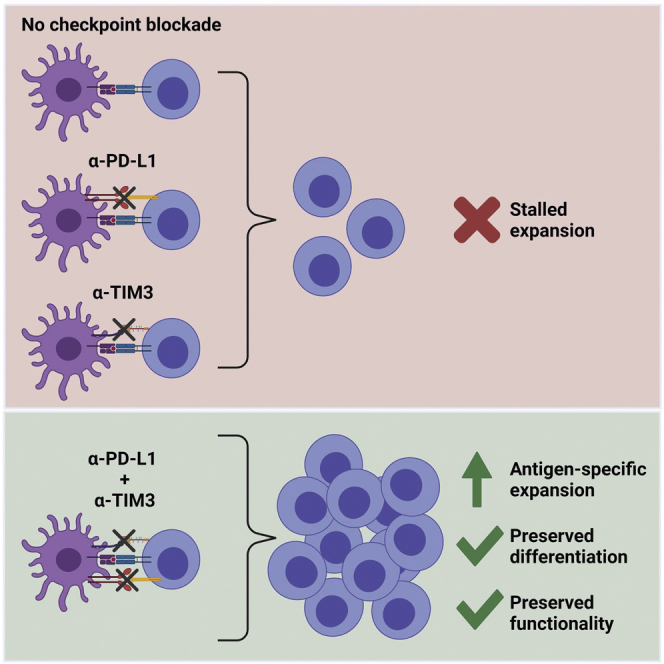

## Teaser

Lak et al. reveal that combined blockade of the immune checkpoints TIM3 and PD-L1 can be leveraged to improve the yield of functional antigen-specific CD8^+^ T cells expanded *ex vivo* for adoptive immunotherapy. This approach represents a readily applicable strategy to improve manufacturing of antigen-specific T cell immunotherapies.

## Introduction

The vast majority of potentially actionable microbial and cancer antigens are major histocompatibility complex (MHC)-bound peptides.^1^^,^^2^ Although it is possible to isolate T cell receptors (TCR) targeting some of these antigens and manufacture TCR-transgenic T cells *ex vivo* followed by their injection into affected individuals, such adoptive immunotherapy approaches face numerous technical hurdles and are currently available only for a minority of relevant cancer antigens.^3^ Several effective strategies rely on *ex vivo* expansion of native antigen-specific T cells, enabling targeting of a vast array of antigens. However, expansion of large numbers of antigen-specific T cells requires repeated antigen exposure (through co-culture with antigen-presenting cells [APCs]) and stimulatory cytokines, potentially leading to T cell dysfunction (terminal effector differentiation and exhaustion) and poor performance after adoptive transfer.^4^, ^5^, ^6^

Alternatively, the endogenous antigen-specific T cell repertoire can be mobilized through systemic administration of antibodies that prevent signaling from inhibitory co-signaling receptors present on the surface of exhausted T cells.^7^^,^^8^ This “immune checkpoint” blockade, most commonly targeting cytotoxic T lymphocyte-associated protein 4 (CTLA-4) and programmed death 1 (PD-1) on T cells (or its corresponding primary ligand, programmed death-ligand 1 [PD-L1]), is now the cornerstone of therapeutic regimens against several types of neoplasia, including advanced melanoma and lung cancer.^9^ Because dysfunctional cancer-reactive T cells often express multiple negative co-signaling molecules, a strategy has been to use combined approaches with the caveat that increased response may come with more immune-related toxicities.^10^ *Ex vivo*-expanded T cells express inhibitory receptors, and PD-L1/PD-L2-silenced antigen-presenting dendritic cells have been shown to improve the expansion and function of antigen-specific T cells *ex vivo*,^11^ providing a solid rationale to leverage immune checkpoint blockade to improve T cell manufacturing for adoptive immunotherapy.

We show here that *ex vivo* expansion of CD8^+^ T cells specific for an oncogenic virus antigen and a tumor-associated antigen (TAA) is enhanced by using combined blockade of PD-L1 and the immune checkpoint T cell immunoglobulin and mucin-containing protein 3 (TIM3), whereas single blockade of either receptor failed to improve T cell expansion. Increased antigen-specific T cell expansion under dual immune checkpoint blockade was not associated with phenotypic and functional evidence of exhaustion or terminal effector differentiation. This was corroborated with single-cell RNA sequencing (scRNA-seq) and V(D)J (variable (V), diversity (D) and joining (J) gene segment) sequencing, which revealed that dual immune checkpoint blockade may affect T cell gene expression in a donor- and clonotype-dependent fashion. Our results show that dual PD-L1/TIM3 blockade during *ex vivo* expansion can yield large quantities of fit human antigen-specific T cells for adoptive immunotherapy.

## Results

### Multiple stimulations are detrimental to antigen-specific T cell expansion *ex vivo*

**Figure 1:**
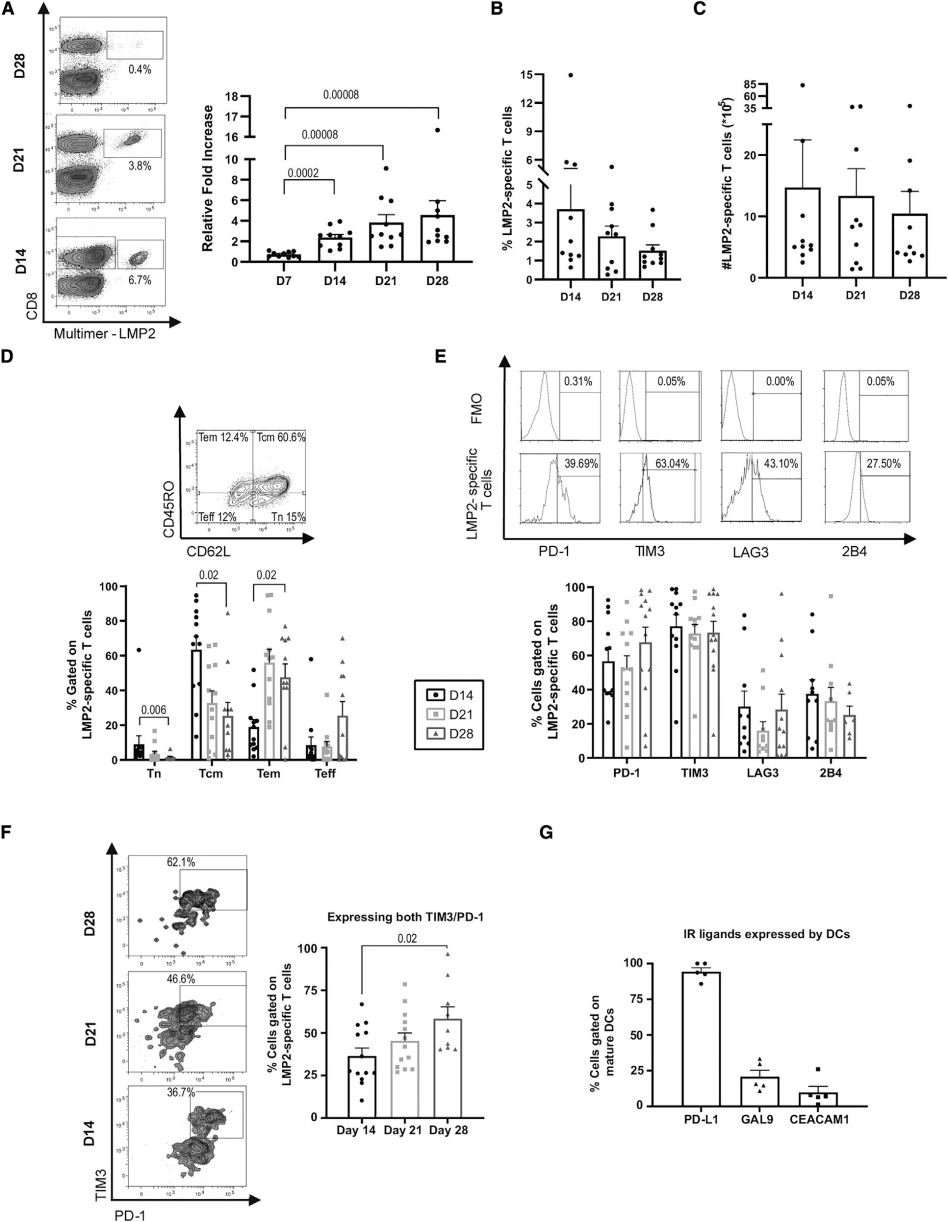
Repeated antigenic encounters *ex vivo* lead to antigen-specific T cell exhaustion (A) Representative fluorescent multimer staining of LMP2_426–434_-specific CD8^+^ T cells (LMP2) (in boxes, with relative abundance indicated as percentages) and total cellular expansion (expressed as fold change relative to culture input, 15 × 106 cells). (B) Percentages (B) as well as (C) calculated number of antigen-specific T cells at the indicated time points (n = 10 different donors). (D) Representative staining to determine the proportions of naive T (Tn) cells, central memory T (Tcm) cells, effector memory T (Tem) cells, and effector T (Teff) cells and the percentage of HLA-A02/LMP2_426–434_ multimer-positive CD8^+^ T cells in these categories at different culture time points (n = 12 different donors). (E) Representative histograms of immune checkpoint expression evaluated relative to fluorescence minus one (FMO) CTRL in HLA-A0201-LMP2_426–434_ (LMP2) multimer-stained T cells and compiled results from 12 different donors. (F) Representative staining of PD-1 and TIM3 co-expression and compiled results from 9–13 different donors. (G) Percentage expression of PD-1 and TIM3 ligands on DCs used to stimulate T cells (n = 5 different donors). Significant p values are indicated. All error bars represent standard error of the mean (SEM).

Antigen-specific T cells were stimulated using monocyte-derived dendritic cells (moDCs) loaded with the peptide latent membrane protein 2 (LMP2)_426–434_ (CLGGLLTMV), an Human leukocyte antigen (HLA)-A0201-restricted antigen from Epstein-Barr virus (EBV). Weekly *ex vivo* stimulation was performed in cytokine-supplemented medium, and antigen-specific expansion was assessed before each stimulation. Although overall T cell expansion progressed after each of the four stimulations (albeit at a limited pace after day 14 and two stimulations), the percentage and absolute number of antigen-specific CD8^+^ T cells, as identified by fluorescent HLA-0201/LMP2_426–434_ multimer, stagnated despite additional stimulation (Figures 1A–1C). This halted growth was matched by a gradual change in phenotype. The predominance of the central memory T (Tcm) cell differentiation profile on day 14 evolved toward an effector memory (Tem) cell and effector T (Teff) cell differentiation phenotype on days 21 and 28 (Figure 1D). These results were anticipated because serial T cell stimulation has been associated with development of Teff cells that gradually lose their capacity to expand and eventually persist after adoptive transfer.^6^ Expression of the inhibitory receptors related to T cell exhaustion (PD-1, TIM3, and, to a lesser extent, LAG3 and 2B4) was substantial on a significant fraction of antigen-specific T cells on day 14 with little modulation over time in culture (Figure 1E). However, the fraction of cells showing dual expression of PD-1 and TIM3 increased with repeated stimulation (Figure 1F), in line with reports linking double expression with severe CD8^+^ T cell exhaustion.^12^, ^13^, ^14^ Phenotyping of moDCs revealed prevalent expression of the corresponding PD-1 and TIM3 ligands, (PD-L1 and CEACAM1/Galectin 9, respectively) (Figure 1G). These results confirm and extend previous data, suggesting that the early and persistent expression of PD-1 and TIM3 by CD8^+^ T cells, along with expression of their ligands by stimulating DCs, may represent a significant hurdle for expansion of antigen-specific T cells for immunotherapy.^12^^,^^13^

### Combination of PD-L1/PD-1 axis and TIM3 blockade significantly increases antigen-specific CD8^+^ T cell expansion

**Figure 2:**
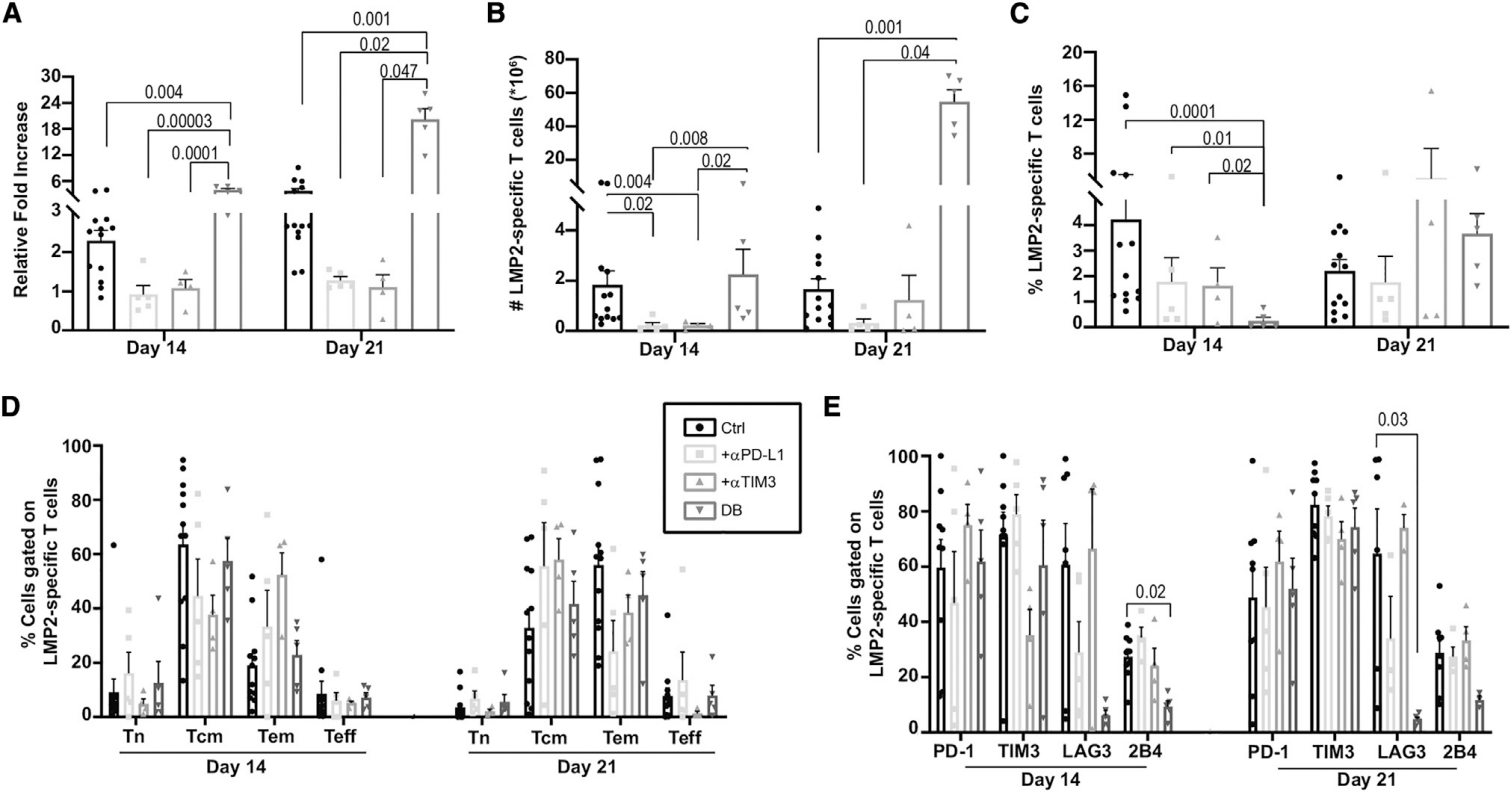
Dual but not single PD-L1 and TIM3 blockade improves T cell expansion (A) Total cell expansion relative to input at the beginning of the culture in function of time and culture condition; no blocking antibodies (CTRL), anti-PD-L1 (α-PD-L1), anti-TIM3 (α-TIM3), or both (double blockade [DB]). (B and C) Absolute count (B) and percentage (C) of HLA-A0201-LMP2_426–434_ (LMP2) multimer-positive T cells under the same conditions and at the same time points. (D and E) T cell differentiation phenotypes (Tn, Tcm, Tem, and Teff cells) (D) and percentages (E) of LMP2 multimer-positive T cells expressing immune checkpoints in the function of culture conditions and time points (n = 4–10 different donors). Significant p values (p < 0.05) are indicated. All error bars represent SEM.

To assess whether immune checkpoint blockade during *ex vivo* expansion may improve antigen-specific T cell yield, anti-PD-L1, anti-TIM3, or both were added to the culture medium at the beginning of culture and with all medium changes. Cell counts on day 14, and even more strikingly on day 21, revealed that the double blockade condition significantly increased T cell expansion relative to the control (no checkpoint blockade) and the single-blockade groups (Figure 2A). This translated into a marked increase in LMP2_426–434_-specific T cell yield under the dual blockade condition, most evident on day 21 of culture (Figures 2B and 2C). In contrast, single blockade of PD-L1 or TIM3 offered no advantage at any time point and even seemed to be detrimental in terms of T cell expansion relative to the control condition. Phenotypic assessment of LMP2_426–434_-specific T cells revealed no statistically significant difference in the percentages of Tcm, Tem, and Teff cells and no difference in PD-1 or TIM3 expression (Figures 2D and 2E). However, the percentage of T cells expressing the inhibitory receptors LAG3 and 2B4 was lower in the combined relative to the control condition. Hence, dual PD-L1 and TIM3 blockade increased T cell growth without conferring phenotypic changes associated with increased T cell dysfunction.

**Figure 3:**
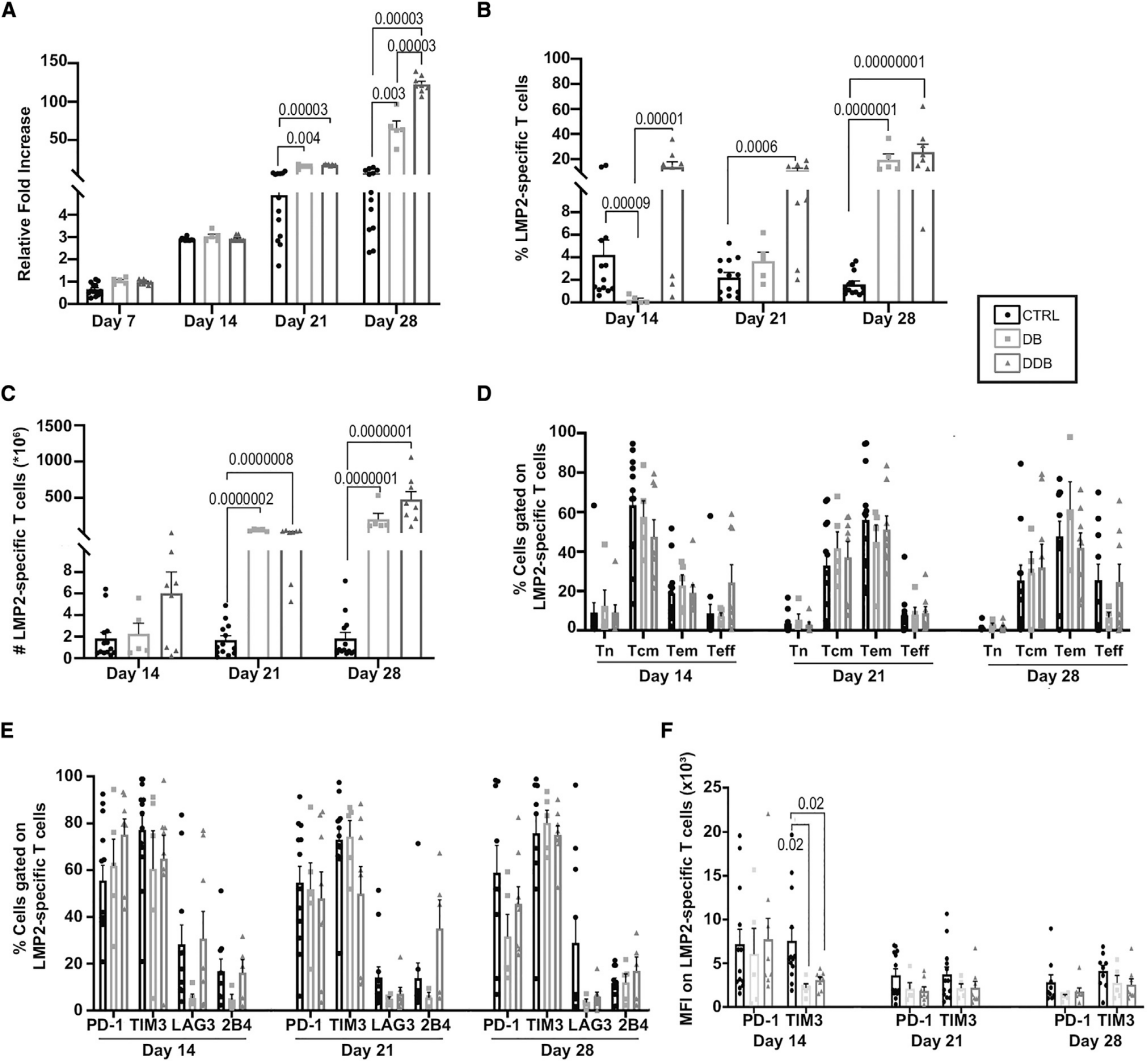
DDB further improves T cell yield (A) Total T cell expansion relative to input on day 0 in the function of time and culture condition; no blocking antibodies (CTRL), double anti-PD-L1 and anti-TIM3 applied at culture initiation (DB), and anti-PD-L1 introduced on day 0 and anti-TIM3 introduced on day 7 (delayed DB [DDB]). (B and C) Percentages (B) and absolute counts (C) of HLA-A0201-LMP2_426–434_ (LMP2) multimer-positive T cells from the same cultures. (D and E) T cell differentiation phenotypes (D) and immune checkpoint expression (E) of LMP2 multimer-positive cells under the same culture conditions and time points (n = 8 different donors). (F) Mean fluorescence intensity (MFI) of PD-1 and TIM3 in function of time in culture. Significant p values (p < 0.05) are indicated. All error bars represent SEM.

It is known that TIM3 has a dual function. TIM3 is transiently upregulated at intermediate levels on activated T cells and confers activation signals.^15^ However, in settings of chronic stimulation in the presence of its ligands, TIM3 inhibits T cell activation and behaves as a *bona fide* immune checkpoint. Accordingly, delayed introduction of single TIM3 blockade on day 7 modestly favored T cell growth (but not antigen-specific T cell yield over the control condition), whereas delayed single PD-L1 blockade did not (Figure S1). We therefore slightly modified our protocol to introduce TIM3 blockade on day 7, a week after the first stimulation (hereafter designated as delayed double blockade [DDB]), with the expectation that it would further improve antigen-specific T cell yield. Compared with the dual blockade started on day 0, DDB marginally increased total T cell expansion on day 21 but more significantly after an additional antigenic stimulation (day 28) (Figure 3A). The DDB approach increased the percentage of LMP2_426–434_-specific T cells at all time points relative to the control condition (no blocking antibodies used), including on day 14, which was not the case when PD-L1 and TIM3 blockade were applied on day 0 (Figure 3B). Absolute antigen-specific counts were also increased under the DDB relative to the control condition (significant on days 21 and 28) (Figure 3C). Exogenous interleukin-2 (IL-2) could be responsible for potentiating T cell expansion in our system,^16^ but the presence or absence of IL-2 in DDB did not consistently affect antigenic T cell yield (Figure S2). The distribution of antigen-specific T cells within memory and effector T cell subsets was similar across conditions, and the fraction of antigen-specific T cells expressing exhaustion markers (PD-1 and TIM3) expression was both highly prevalent (Figures 3D–3F) and similarly distributed across memory and effector T cell subsets in both conditions (Figure S3). The surface density of PD-1 and TIM3, as evaluated by mean fluorescence intensity (MFI), also remained similar across conditions at later time points, but TIM3 MFI was lower in the context of double blockade on day 14, perhaps conditioning the improved expansion occurring subsequently. Hence, despite markedly increased expansion, double blockade did not increase effector differentiation or exhaustion marker expression. Independent cultures using isotype control antibodies confirmed the specific effects of anti-PD-L1 and anti-TIM3 antibodies on antigen-specific T cell expansion (Figure S4), and delaying the introduction of anti-TIM3 and anti-PD-L1 on day 7 of culture did not favor T cell expansion (Figure S5). We conclude that combined blockade of the PD-L1/PD-1 axis and TIM3 can be incorporated in *ex vivo* cultures to increase antigen-specific CD8^+^ T cell yield for adoptive immunotherapy without altering T cell phenotypes.

### Double immune checkpoint blockade generates functional antigen-specific T cells

**Figure 4:**
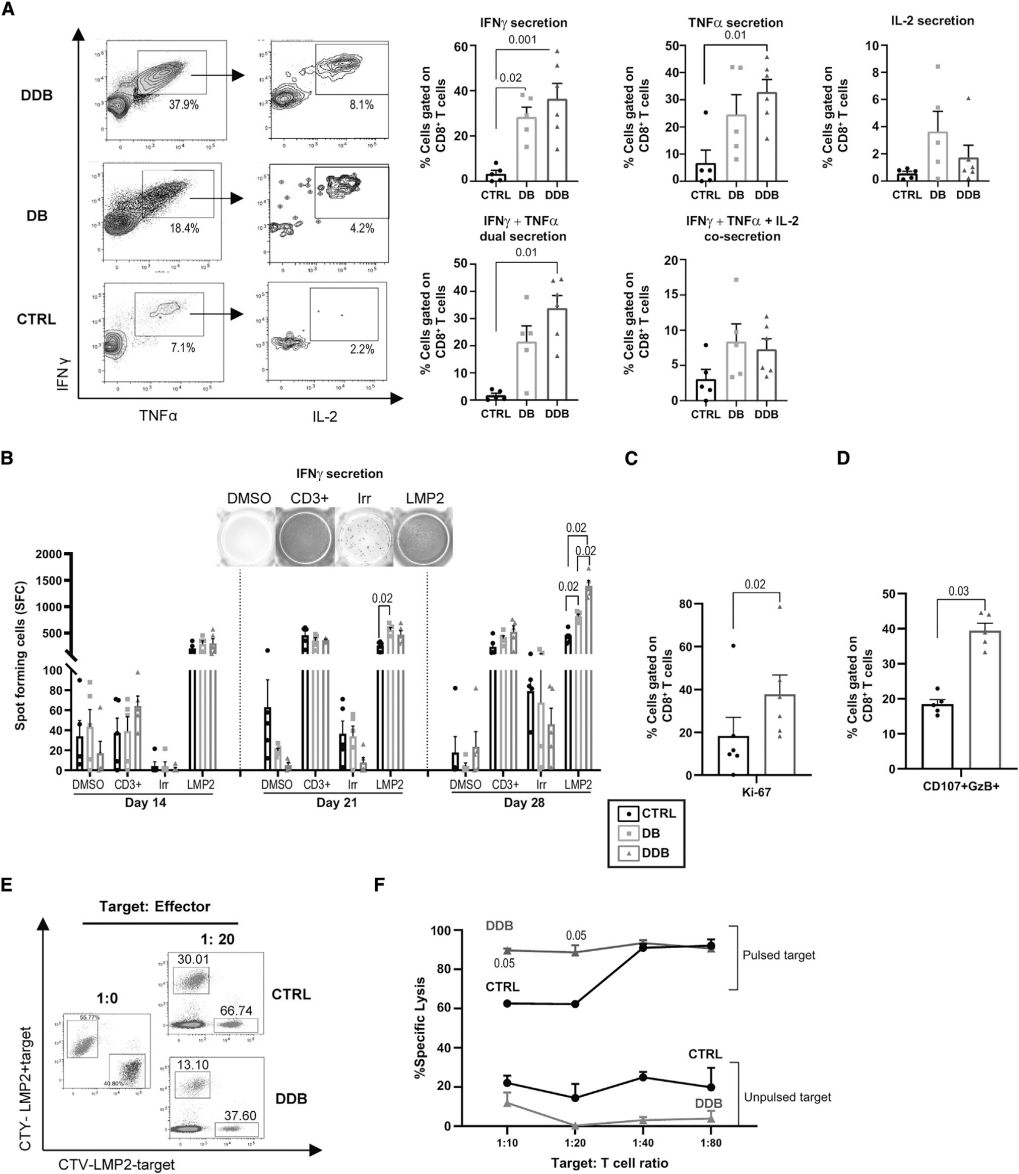
DDB expands a high proportion and number of functional antigen-specific T cells (A) Representative dot plots showing intracellular cytokine secretion after LMP2_426–434_ antigenic re-stimulation on day 28 and compiled results from 5–6 independent donor cultures, demonstrating the percentage of CD8^+^ T cells secreting IFNγ, TNF-α, IL-2, and multiple cytokines; expansion with no blocking antibodies (CTRL), anti-PD-L1 and anti-TIM3 antibodies introduced at the beginning of the culture (DB), or both antibodies but anti-TIM3 introduced on day 7 (DDB). Percentages of events in boxes are indicated for double (IFNγ and TNF-α, left) and triple (IFNγ, TNF-α, and IL-2, right) cytokine-expressing cells. (B) IFNγ ELISpot results using 50,000 cells per condition, harvested from the cultures at the indicated time points and using the following stimulation conditions: vehicle only (DMSO), anti-CD3 (CD3, positive CTRL), irrelevant (Irr) peptides, and LMP2_426–434_ peptide (LMP2). (C and D) Percentage of Ki-67 staining on day 28 among CD8^+^ T cells from CTRL versus DDB cultures (n = 5 different donors) (C) and co-expression of surface CD107a and intracellular granzyme B (GzB) (D) as a surrogate for degranulation after LMP2_426–434_ exposure. (E and F) Fluorescence-based cytotoxicity assay (Cell Tracer Yellow [CTY] or Cell Tracer Violet [CTV]) showing loss of targets loaded (LMP2+) or not loaded (LMP2−) with the LMP2_426–434_ antigen (numbers indicate percentages of total events) (E) and compiled results at different target:T cell ratios (from 3 different donors) (F). Significant p values are indicated, and error bars indicate SEM.

Dual PD-L1 and TIM3 blockade increases antigen-specific CD8^+^ T cell expansion in culture without altering T cell phenotypes, suggesting comparable functionality. Intracellular cytokine secretion measurements and enzyme-linked immune-spot (ELISpot) assays on day 28 confirmed that a higher proportion of T cells was reactive upon LMP2_426–434_ peptide re-exposure under the DDB condition relative to the control (no checkpoint inhibition) (Figures 4A and 4B). This was also generally the case when DDB was compared with double immune checkpoint blockade administered on day 0 (statistically significant in ELISpot data on day 28). Based on these findings, we concentrated our functional characterization by comparing the control condition with DDB, which had surpassed double blockade (DB) in terms of T cell expansion and function and was thereafter selected to be our reference to test the effect of dual immune checkpoint blockade on antigen-specific CD8^+^ T cells for the rest of the study. As further indication of increased functionality, a greater fraction of antigen-specific T cells expanded under the DDB condition relative to the control expressed the proliferation marker Ki-67, and upon peptide re-exposure, more DDB-exposed T cells displayed evidence of cytotoxic potential (surface CD107a and intracellular granzyme B expression) (Figures 4C and 4D). This was corroborated by cytotoxicity assays showing that T cells from the DDB condition were highly effective, especially at low effector:target ratios (Figures 4E and 4F). In all our assays, combined PD-L1 and TIM3 blockade did not lead to increased non-specific (off-target or spontaneous) cytokine release or cytotoxicity. Thus, dual immune checkpoint inhibition expands T cell products with specific and augmented antigen reactivity.

### PD-L1/TIM3 blockade imparts no consistent gene expression signatures to expanded antigen-specific CD8^+^ T cells

**Figure 5:**
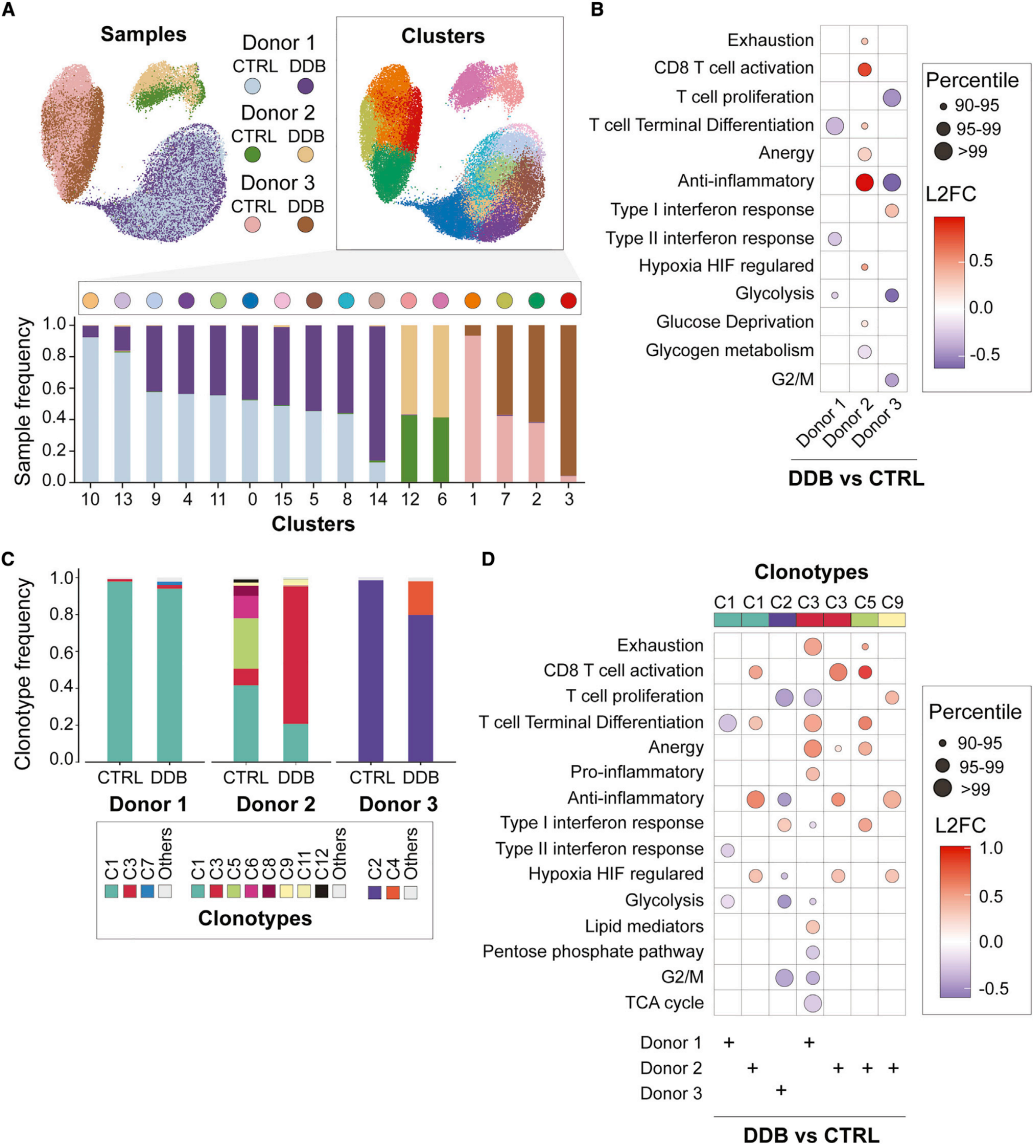
scRNA-seq of antigen-specific T cells after double immune checkpoint inhibition (A) T-stochastic neighbor embedding (t-SNE) of normalized single-cell gene expression after dimensionality reduction from control (CTRL) and DDB conditions, color coded by donor and experimental condition on the left and by cluster on the right. The barplot represents the percentage of each sample (colored as above left) in each cluster labeled on the x axis and color-coded at the top of the graph. (B) Dot plot representing the change in expression of genes related to pathways of interest under the DDB condition compared with the CTRL condition. The color of the dots represents the log2 fold change of the genes in the pathway, and the size of the dot is representative of the percentile ranking of the comparison in random gene sets (Materials and methods). (C) Clonotype frequencies under the CTRL and DDB conditions for each donor. (D) Similar to (B), comparing cells of selected clonotypes/affected individuals between the two experimental conditions.

To gain more insight into the biological effects of double immune checkpoint blockade on antigen-reactive T cells, we submitted sorted day 28 multimer-positive T cells from three donors to paired transcriptome and TCR alpha-beta scRNA-seq. Gene expression was compared between donors, conditions (no checkpoint blockade versus DDB), and clonotypes. Global gene expression patterns across the three donors revealed strong donor-specific clustering with no significant multidonor contribution to any of the identified clusters (Figure 5A). We thus focused on the effect of DDB on gene expression of antigen-specific T cells in each donor using published human T cell gene sets.^17^ This enabled a detailed assessment of T cell activation, proliferation, terminal differentiation, exhaustion, and metabolism-associated gene expression because these processes are likely to be affected by DDB. Overall, when all T cells from each condition were compared, no consistent and common changes in gene expression signature could be identified across all donors (Figure 5B). We next evaluated whether clonotype-specific signatures could be identified. V(D)J sequencing revealed that LMP2_426–434_-specific T cells were oligoclonal in all donors and conditions (1–4 clones representing more than 80% of all cells; Figure 5C and Table S1). Most dominant clonotypes were shared between experimental conditions (but in some instances at different frequencies), and a few clonotypes were shared between donors (e.g., clonotype 1 and 3, common to donors 1 and 2; Table S1). We next analyzed the expression of several pathways in the abundant clonotypes, defined as those represented by at least 10 cells and representing at least 1% of the repertoire under the DDB and control conditions from the same donor. Similar to comparisons involving all clonotypes, no consistent pattern was found across donors when comparing the DDB and control condition on a per-clonotype basis (Figure 5D). Our data unveiled divergent gene expression patterns for one clonotype (clonotype 3) that was shared by donors 1 and 2. Relative to its counterpart under the control condition, donor 1 clonotype 3 under the DDB condition expressed higher levels of genes related to T cell exhaustion/differentiation and had a lower expression of genes associated with T cell proliferation. The same clonotype under the DDB condition from donor 2 had, on the contrary, increased expression of genes associated with T cell activation without any transcriptional changes related to T cell dysfunction (T cell exhaustion, terminal differentiation, low proliferation). Although obtained from a limited number of cells and donors, these results suggest that donor- rather than clonotype-related features may determine T cell outcomes after DDB.

**Figure 6:**
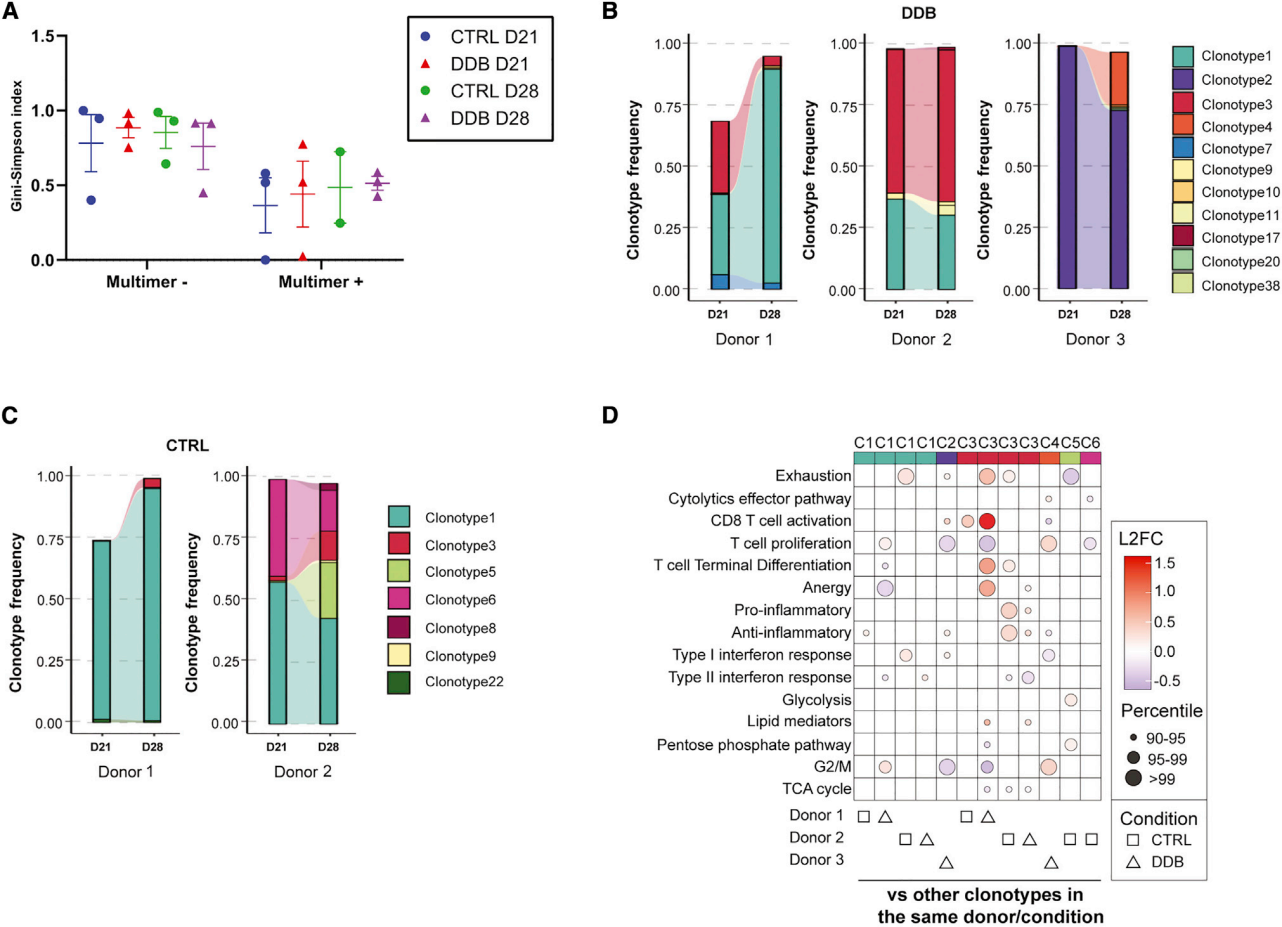
Effect of DDB on clonal diversity and stability in time (A) Estimate of TCR repertoire diversity using the Gini-Simpson index among HLA-multimer-negative and -positive T cells on days 21 and 28. (B and C) Clonal hierarchy and clonal relatedness among HLA-A0201-LMP2_426–434_ multimer-positive T cells between days 21 and 28 using the day 28 repertoire as a reference under the DDB (B) or CTRL condition (C), as determined by bulk CDR3 sequencing. The CTRL condition in donor 3 was not assessed because of poor RNA quality on day 28. (D) Dot plot representing the change in expression of genes related to pathways of interest when comparing a clonotype of interest in a donor/experimental condition with all other clonotypes in the same donor/condition. The clonotypes selected are those whose abundance vary by more than 20% between days 21 and 28.

To assess whether clonotype-specific transcriptional signatures may affect their expansion and clonal hierarchy within the cultures, we used bulk mRNA collected from sorted multimer-negative and -positive CD8^+^ T cells on day 21 and 28 from the same donors. RNA of suitable quality was obtained for 23 of a potential of 24 samples (the multimer-positive fraction of the control condition from donor 3 had to be excluded) and subjected to complementary-determining region 3 (CDR3) region sequencing of the TCR beta chain (TCRβ). Day 28 multimer-positive T cell CDR3 sequencing very well matched paired V(D)J sequencing of single cells (Figure S6), confirmed the oligoclonality of the multimer-positive T cells on days 21 and 28, and revealed that TCR repertoire diversity in the multimer-negative T cells was not affected by DDB (Figure 6A). The multimer-positive and -negative fractions had limited overlap (Figure S7), suggesting that sorting effectively separated most CD8^+^ antigen-specific cells from the rest of the T cells in culture. Clonotype hierarchy among multimer-positive T cells on day 21 and day 28 showed no consistent pattern of evolution in DDB relative to the control condition (Figures 6B and 6C). However, the proportion of certain clonotypes in these fractions (DDB and control) varied significantly (>20%) between days 21 and 28. This was notably the case for clonotype 3 from donor 1 under the DDB condition, which declined markedly from day 21 to day 28, whereas this clonotype’s frequency changed only slightly between days 21–28 of DDB exposure in donor 2 (Figure 6B). We then explored whether the clonotypes with altered abundance between day 21 and day 28 in the same donor and condition displayed a specific transcriptional profile on day 28. Under the DDB condition, clonotype 3 of donor 1, which showed decreased abundance from day 21 to day 28, had a higher expression of genes related to T cell activation, differentiation, and exhaustion relative to other clonotypes from the same donor under the same condition (Figure 6D). Although less striking, we also noticed a weak trend of increased expression of genes related to exhaustion in clonotypes with decreased abundance on day 28 and a rise in the expression of genes related to proliferation in clonotypes with increased abundance on day 28, irrespective of experimental conditions (Figure 6D). These data show that DDB has a limited effect on the clonal diversity of the expanded T cells over time. The proportion of the various clonotypes in time can nonetheless fluctuate under both culture conditions, and the gene expression signatures on day 28 offer possible explanations for such fluctuations. These results suggest that DDB confers no consistent transcriptional features to expanded antigen-specific CD8^+^ antigen-specific T cells but may alter activation/dysfunction and cell cycle-related processes in a clonotype- and donor-dependent fashion.

### The benefits of dual PD-L1 and TIM3 blockade extend to TAA-specific T cells but not to all T cell manufacturing protocols

**Figure 7:**
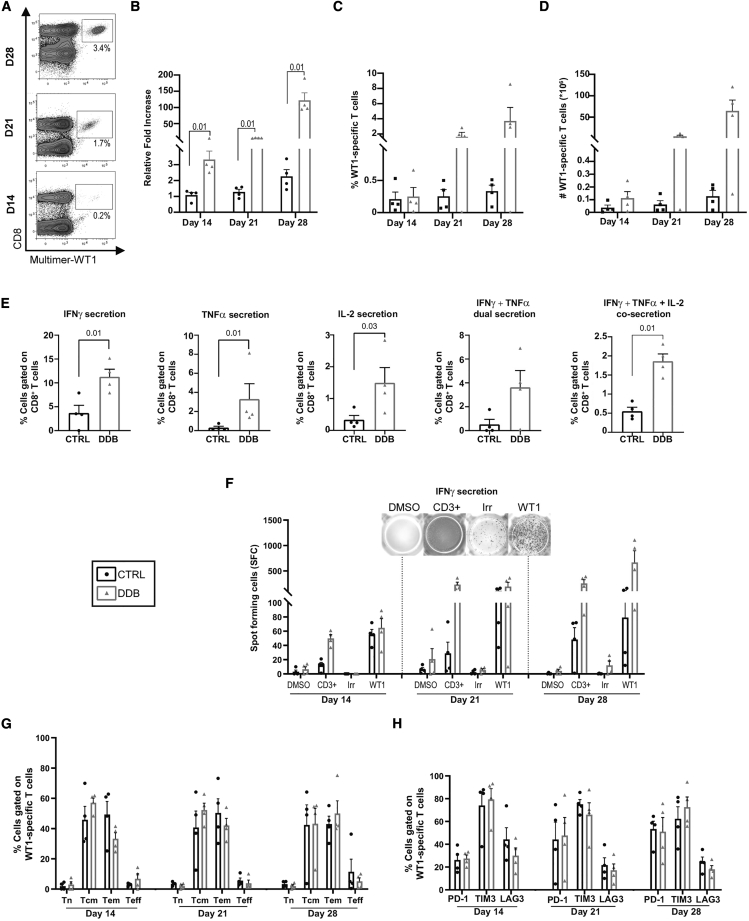
Improved expansion of functional WT1-specific T cells with DDB (A) Representative HLA-A0201-WT1_37–45_ multimer staining (WT1) of CD8^+^ T cells (boxes) with relative frequency in the culture, as indicated by percentages. (B–D) Cell expansion expressed as fold increase relative to cell input at the beginning of the culture (15 × 106) under CTRL (no blocking antibodies) or anti-PD-L1 and anti-TIM3 DDB) conditions (B) as well as percentages (C) and absolute numbers (D) of WT1-specific T cells at different time points in culture. (E and F) Proportion of intracellular cytokine expression (IFNγ, TNF-α, IL-2, and multiple cytokines) after WT1 exposure on day 28 of culture in CD8^+^ cells (E) and IFN-γ ELISpot results on days 14, 21, and 28 of culture (F) after exposure to vehicle alone (DMSO), peptides not used in the culture (Irr) as negative CTRL, anti-CD3ϵ (positive CTRL), and the targeted peptide (WT1). (G and H) T cell differentiation (Tn, Tcm, Tem, and Teff cells) (G) and percentage of immune checkpoint surface expression (H) on WT-1 specific T cells. Four different donors; p values are indicated; error bars indicate SEM.

The EBV-derived LMP2_426–434_ antigen stimulates a memory T cell repertoire in more than 90% of adults.^18^ *Ex vivo* expansion of naive T cells is generally considered more challenging for several reasons, such as size of the repertoire and amount of stimulation required. We sought to determine the effect of dual PD-L1/TIM3 blockade on priming and expansion of naive T cells. It has been shown previously that CD8^+^ T cells specific against Wilm’s tumor 1 (WT1)-derived peptides, a clinically relevant TAA, are phenotypically naive in healthy individuals^19^ but can be expanded from a majority of such donors.^20^^,^^21^ Volunteer donor T cells were stimulated with an HLA-0201-restricted WT1 peptide (WT1_37–45_) using the same stimulation/expansion conditions for LMP2_426–434_-specific T cells. WT1-specific T cells expanded, following a similar pattern as LMP2_426–434_-specific T cells (Figures 7A–7C). The expansion, percentages, and numbers of antigen-specific cells as well as their cytokine secretion were improved under the DDB condition relative to cultures without immune checkpoint blockade, again without altering T cell differentiation patterns or exhaustion marker expression (Figures 7D–7H). We conclude that our results obtained with LMP2_426–434_ extend beyond virus-specific memory T cell expansion and that double PD-L1/TIM-3 immune checkpoint blockade can improve expansion of functional TAA-specific CD8^+^ T cells from naive repertoires. Expansion of antigen-specific T cells requires strong and repeated stimulation that is conducive to immune checkpoint expression. We next sought to assess whether dual checkpoint inhibition would be beneficial in the setting of another clinically compliant system to expand polyspecific virus-reactive cells from relatively abundant memory T cell repertoires.^22^, ^23^, ^24^ Through use of peptide libraries, IL-7 and IL-4, these short cultures (9–14 days) expand T cell lines with limited expression of immune checkpoints. We found that DDB had no discernible effect in terms of T cell expansion, differentiation, and antigen reactivity in this setting (Figure S8). Similarly, use of DDB during chimeric antigen receptor (CAR) T cell generation did not improve CAR T cell yield despite robust expression of PD-1 and TIM3 and their ligand PD-L1 and Galectin 9 by the T cells in the culture (Figure S9). Agonistic anti-CD3 and anti-CD28 stimulation and IL-7, IL-15, and IL-2 cytokine supplementation were used on purpose to provide strong activation signals. These results suggest that the benefits of dual immune checkpoint blockade heavily depend on culture duration as well as repeated stimulation rather than simple immune checkpoint expression.

## Discussion

Stimulation and expansion of antigen-specific T cells for adoptive immunotherapy is an attractive strategy to target a wide variety of antigens. However, T cell expansion is limited by expression of inhibitory receptors and development of T cell dysfunction. We confirmed and extended previous studies revealing that immune checkpoint receptors are expressed on repeatedly activated T cells and that their corresponding ligands can be present on APCs used *ex vivo*. Co-expression of PD-1 and TIM3 has been used to describe highly exhausted CD8^+^ T cells, and combined blockade of these receptors in murine models has been shown previously to improve tumor control relative to single blockade.^12^^,^^13^^,^^25^ In humans, dysfunctional tumor-infiltrating lymphocytes (TILs) could be reinvigorated through combined TIM3 and PD-1 blockade *in vitro*, providing a rationale to incorporate dual blockade in T cell manufacturing.^26^ We show that antibody-mediated immune checkpoint blockade targeting PD-1 or TIM3 alone is insufficient to improve CD8^+^ T cell expansion, whereas the combination improves T cell expansion and antigen-specific reactivity. Intracellular cytokine staining and ELISpot revealed increased functional antigen-specific T cell yield after DDB but to different degrees relative to the control, highlighting the difference in sensitivity between the two assays^27^^,^^28^ and underscoring how difficult it is to quantitatively define the beneficial effect of dual checkpoint blockade. Our data contrast with a previous study where PD-1 blockade alone was able to restore human T cell functionality against EBV^+^ lymphoma cell lines *ex vivo*,^29^ stressing the importance of adapting checkpoint blockade approaches for every culture system. We demonstrate this by showing that immune checkpoint blockade has different efficacy in various clinically relevant processes. Rapidly generated virus-specific T cell lines and CAR T cell generation did not benefit from DDB in our hands. These two T cell products differed in the type of stimulation they received, and we expected a benefit in CAR T cells, where strong stimulation is associated with high expression of immune checkpoints and their ligands (which themselves can have inhibitory functions).^30^ As suggested by our data, the benefit of immune checkpoint blockade may only become apparent after repeated stimulation in long-term culture. Hence, *ex vivo* T cell expansion protocols requiring extended culture duration, such as those used in TIL therapy, may be the next candidates for testing immune checkpoint blockade during manufacturing.^31^ Our data confirm the synergistic potential of immune checkpoint blockade and the particular relevance of TIM3 and PD-1 as inhibitory receptors in CD8^+^ T cells.^13^ The reasons for this synergistic effect might be mobilization of different signaling intermediates by the two receptors. PD-1 relies on recruitment of Src homology region 2 domain-containing phosphatase (SHP) to mediate its effects (as for several other negative co-stimulatory molecules), and TIM3 uses BAT3/BAG6 and FYN Proto-Oncogene (FYN) for its stimulatory and inhibitory effects, respectively. We reasoned that delaying TIM3 blockade by 1 week after the start of the culture would be beneficial, given previous experimental evidence supporting that TIM3 provides activation signals after the first T cell stimulation.^32^^,^^33^ This contrasts with PD-1, which conveys inhibitory signals in exhausted T cells and early after activation,^34^ justifying use of anti-PD-L1 at culture initiation. However, single blockade of PD-L1 or TIM3 appeared to decrease T cell yield when started at culture initiation. We explored this through complementary experiments and observed that delaying the single blockade of TIM3 slightly increased T cell expansion, which was not the case with delayed PD-L1 inhibition, in accordance with currently accepted notions regarding these receptors. However, delaying anti-TIM3 and anti-PD-L1 did not have significant effects. We realize that several refinements may be required to fully leverage the potential agonistic/antagonistic effects of TIM3 on T cell activation and expansion and determine the optimal timing of anti-PD-L1/PD-1 blockade. Other modifications could also be readily tested, such as blockade of additional inhibitory receptors alone and in combination as well as different cytokine combinations that may influence PD-1 and TIM3 expression^35^^,^^36^ and provide synergistic effects, which was not revealed for IL-2 in our system. Our work provides a new strategy for antigen-specific T cell expansion and offers biological insights on the effects of PD-L1/PD-1 axis and TIM3 blockade in human antigen-specific T cell clonotypes submitted to multiple antigenic stimulations.

Our data support that sustained dual PD-L1/TIM3 blockade through several rounds of antigenic stimulation provides ongoing benefits without exacerbating T cell dysfunction or curbing expansion, as inferred by previous studies of using inhibitory receptor gene deletion.^37^ This was assessed through phenotyping, functional assays, and gene expression in single cells. Our results indicate that antigen-specific CD8^+^ T cells expanded under combined TIM3/PD-L1 blockade are functional, display similar proportions of memory and effector subsets as defined phenotypically, and show a similar pattern of immune checkpoint expression across memory and effector subsets and conditions at the end of the culture. This suggests that dual immune checkpoint blockade may expand early memory-expressing immune checkpoints^12^^,^^38^ and late memory T cells. Likewise, clonal composition in the cultures was found to be generally similar between DDB and control conditions and stable in time. It has been reported that immune checkpoint blockade *in vivo* can shape the T cell repertoire.^39^, ^40^, ^41^ Thus, from a perspective of T cell therapy, it was essential to define whether an intervention during *ex vivo* T cell expansion alters the clonal identity of the cellular product. Although we did not observe consistent effects of PD-L1/TIM3 blockade on the T cell transcriptome or clonotype distribution, altered transcriptional profiles in a clonotype- and donor-dependent manner nonetheless suggests that immune checkpoint blockade in adoptive T cell therapy may require personalization. Although requiring confirmation, our data on clonal dynamics and transcriptomes offer insights about the effects of immune checkpoint blockade on human antigen-specific CD8^+^ T cells. The divergent fates of a shared clonotype between two donors infer that TCR affinity is not a preponderant factor dictating cellular responses to PD-1 and TIM3 blockade, contrasting with previous observations with anti-CTLA-4 and PD-1 blockade in mice.^42^ Further work will be required to understand why some clonotypes in certain individuals may be more susceptible to differentiation/exhaustion after immune checkpoint blockade at different time points. Likely factors that may influence clonal dynamics during/after T cell culture include previous activation/proliferation history, differentiation status at the beginning of checkpoint blockade, and pre-expansion abundance. Our data suggest that, even after 28 days in culture, the antigen-specific T cell population is highly functional, with a third of T cells expressing Tcm cell markers. Although such early memory T cells express inhibitory receptors and show evidence of exhaustion, evidence suggests that this is reversible.^43^ Hence, we surmise that antigen-specific T cells generated in high numbers after dual immune checkpoint blockade could expand more after transfer and likely respond to further immune checkpoint blockade administered *in vivo*.

We conclude that dual PD-L1/TIM3 blockade is a readily applicable strategy to improve functional antigen-specific CD8^+^ T cell expansion *ex vivo* for adoptive immunotherapy.

## Materials and methods

### Donors and cellular procurement

Peripheral blood mononuclear cells (PBMCs) from HLA-A0201-expressing volunteer donors were isolated using Ficoll-Hypaque gradient (STEMCELL Technologies, Vancouver, BC, Canada) from fresh whole blood (collected by venipuncture) or leukoreduction system chambers (LRSCs) provided by Héma-Québec as described previously.^24^^,^^44^ All donors provided written informed consent, and all experiments were approved by the Héma-Québec and Hôpital Maisonneuve-Rosemont Research Ethics Committees. Recovered PBMCs were used immediately for experiments or cryopreserved for future use in 90% fetal bovine serum and 10% dimethyl sulfoxide (DMSO).

### Dendritic cells differentiation and antigen pulsing

Monocyte isolation and DC differentiation were performed as described previously.^4^ Briefly, monocytes were obtained using the adherence method, where PBMCs were plated in adherent plastic plates (Sarstedt, Nümbrecht, Germany) in medium (X-Vivo 15 medium, Lonza, Basel, Switzerland) supplemented with 5% human serum, 2 mM L-glutamine and 1 mM sodium pyruvate (Thermo Fisher Scientific, Waltham, MA, USA), 1,000 U/mL (100 ng/mL) IL-4, and 800 U/mL (50 ng/mL) Granulocyte-macrophage colony-stimulating factor (GM-CSF) (both from STEMCELL Technologies) and incubated in a CO_2_, 37°C incubator for 7 days. On day 4, the medium was replaced with fresh medium supplemented with IL-4 and GM-CSF. On day 7, DCs were matured with maturation medium containing 1,000 U/mL (100 ng/mL) IL-4, 800 U/mL (50 ng/mL) GM-CSF, 10 ng/mL tumor necrosis factor alpha (TNF-α) (STEMCELL Technologies), 1 μg/mL PGE2 (Sigma), 10 ng/mL IL-1β (Feldan, Quebec City, QC, Canada), and 100 ng/mL IL-6 (Miltenyi Biotec, Bergisch Gladbach, Germany) and loaded with the desired peptide (1 μg/mL LMP2_426–434_, [CLGGLLTMV] or 1 μg/mL WT1_37–45_ [VLDFAPPGA], both from JPT Peptides (Berlin, Germany). Last, DC medium was supplemented with interferon γ (IFN-γ; 1,000U/mL, Feldan) for the last 24 h of maturation.

### Antigen-specific T cell expansion and rapid polyspecific T cell line generation

Antigen-specific T cells were stimulated using a clinically compliant protocol as described previously^4^ (ClinicalTrials.gov: NCT03091933) from 15 × 106 PBMCs and expanded through multiple weekly stimulations using irradiated (40 Gy) autologous, peptide-loaded, monocyte-derived DCs at a 1:10 (DC:PBMC) ratio in a G-Rex6 well plate vessel (Wilson Wolf Manufacturing, New Brighton, MN, USA). Our complete T lymphocyte culture (CTL) medium (Advanced RPMI 1640, 10% human serum, 1× L-glutamine [Thermo Fisher Scientific]) was supplemented with the following cytokines: week 1: IL-21 (30 ng/mL) and IL-12 (10 ng/mL) (both from Feldan); week 2: IL-21, IL-2 (100 U/mL), IL-7 (10 ng/mL), and IL-15 (5 ng/mL) (STEMCELL Technologies); subsequent weeks: IL-2, IL-7, and IL-15. Medium, including cytokines, was refreshed every 3–4 days, and antigen re-stimulation was done every week with peptide-loaded, monocyte-derived DCs. Cell concentration was adjusted to a 1:10 ratio each week. When indicated, cultures were supplemented with 20 μg/mL of anti-PD-L1-blocking monoclonal antibody (mAb) (29E.2A3, Bio X Cell, Lebanon, NH, USA) or/and 10 μg/mL of anti-TIM3-blocking mAb (F38-2E2, BioLegend, San Diego, CA, USA) with all medium changes. The respective isotype antibodies, mouse immunoglobulin G1 (IgG1; Bio X Cell, MOPC-21) and InVivoMAb mouse IgG2b (Bio X Cell, MPC-11), were used at the same concentration where indicated. All cell cultures were performed in monitored incubators (37°C in 5% CO_2_ and 5% air humidity). Cell viability and cell counts were assessed by the Countess automated cell counter (Invitrogen/Thermo Fisher Scientific) using trypan blue (Invitrogen) at a 1:1 ratio with the cellular suspension in cell counting chamber slides (Invitrogen, C10283). The polyspecific virus-reactive T cell lines were generated as described with slight modification.^24^^,^^44^ Briefly, 3 × 106 donor PBMCs were exposed to a peptide library covering the entire EBV LMP2 protein (JPT Peptides) and placed in G-Rex24 well plate vessels in T cell medium enriched with IL-7 (10 ng/mL) and IL-4 (400 U/mL). Anti-PD-L1 was introduced on day 0, and anti-TIM3 was introduced at the first half-medium change (day 5), both at the same concentration as mentioned above. On day 9, T cell counts and full-medium changes (that included blocking antibodies) were performed, and the cultures were terminated on day 12.

### CAR T cell generation

The second-generation CAR (CD28 co-stimulation domain) engineered to express a reported gene (non-signaling truncated nerve growth factor receptor [NGFR]) was a kind gift from Jonathan L. Bramson (McMaster University, Hamilton, ON, Canada). Packaging in lentiviruses and transduction were performed as described previously^45^ with slight modifications. Briefly, HEK293T cells were cultured in high-glucose-containing Dulbecco’s modified Eagle’s medium (DMEM; Lonza) supplemented with 10% fetal bovine serum (FBS; Gibco) and 1× L-Glutamine (Gibco) at 37°C and 5% CO_2_. Low-passage HEK239T cells (p12) were plated in 100-mm culture dishes (3 × 106/dish) and transfected after 24 h of culture with 8 μg of packaging plasmid (psPAX2), 4 μg of vesicular stomatitis virus (VSV) envelope (pMD2.G), and 13 μg of CAR-CD19 plasmid (pCCL-CD19). Plasmids were combined in FBS-free OPTI-MEM medium (Gibco) to which TransIT-LT1 (MirusBio) transfection reagent was added. The mixture was incubated at room temperature for 30 min and added dropwise on the culture dishes. Culture medium was changed 24 h after transfection, and viral supernatant was harvested 48 h and 72 h after transfection. Viral supernatant was first passed through a 0.45-μm pore polyvinylidene fluoride (PVDF) Millex-HV filter (Millipore) and then concentrated using Amicon Ultra-15 centrifugal filter units (Millipore Sigma). T cells were isolated from PBMCs of 4 different donors using the EasySep Human T cell Enrichment Kit (STEMCELL Technologies). T cells were plated in 96-well plates (100,000/well) and stimulated with 5 μg/mL of pre-coated purified No azide/low endotoxin (NA/LE) mouse anti-human CD3 (clone OKT3, BD Biosciences) and 1 μg/mL of anti-human CD28 (clone 28.2, eBioscience) on day 0. Cells were cultured in complete CTL medium supplemented with 100 U/mL IL-2 (Miltenyi) and 10 ng/mL IL-7 and 5 ng/mL IL-15 (both from PeproTech) and replenished with every medium change. Anti-PD-L1 was added on day 0 and anti-TIM3 on day 4 at the concentrations stated above, and both were replenished with every medium change. T cells were transduced on day 3 with 10 μL of freshly harvested viral supernatant in the presence of 8 μg/mL Polybrene and 100 μL of complete CTL medium containing the previously mentioned cytokines, and blocking antibodies were added on day 4. Medium changes were performed on day 7 and day 10.

### Flow cytometry

The phenotype of mature DCs was assessed by cell surface expression of the following markers (antibody clone in parenthesis): CD80 (L307.4), CD86 (2331, FUN1), HLA-ABC (W6-32), CD11c (3.9), and CD19 (HIB19) (all from BD Biosciences, Franklin Lakes, NJ, USA) and HLA-DR (LN3) and CD83 (HB15e) (both from Invitrogen). To detect antigen-specific CD8^+^ T cells, up to 10^6^ cells were suspended in phosphate-buffered saline (PBS) plus 2% FBS and stained with the appropriate allophycocyanin-labeled MHC-Dextramer A∗0201 (Immudex, Copenhagen, Denmark) for 10 min in the dark at 4°C. For additional cell surface markers and phenotyping, cells were stained with the following antibodies: CD3 (SKY7), CD3 (UCHT1), CD8 (SK1), CD45RO (UCHL1), CD45RA (5H9), CCR7 (150503), LAG3 (T47.530), Galectin 9 (9M1-3), and CD4 (RPA-T4) (all from BD Biosciences); CD62L (DREG-56), TIM3 (F38-2E2), PD-1 (EH12.2H7), KLRG1 (2F1/KLRG1), CD57 (HCD57), NGFR/CD271 (ME20.4), and PD-L1 (29E.2A3) (from BioLegend); and CD8 (RPA-T8) and CD244 (eBioDM244) (from Thermo Fisher Scientific). Staining was performed at room temperature (RT) in the dark for 30 min. Intracellular staining was done with the Foxp3/Transcription Factor Staining Buffer Set as recommended by the manufacturer (eBioscience/Thermo Fisher Scientific). Before the fixation step, up to 10^6^ cells were stimulated to produce cytokines with the indicated peptide (0.5 μg/mL) (test condition) or phorbol 12-myristate 13-acetate (PMA) (50 ng/mL)-ionomycin (500 ng/mL) (Sigma-Aldrich, St. Louis, MO, USA) (positive control) and a pool of irrelevant peptides (from the cytomegalovirus pp65 protein; PepMix Human Cytomegalovirus - strain AD169 [HCMVA], JPT Peptides) for 4 h at 37°C. Cells were suspended in CTL medium plus brefeldin A (BioLegend) to block secretion of cytokines during the stimulation period. Cells were then harvested and stained for cell surface markers, including CD3, CD4, and CD8, at 4°C for 20 min. Next, fixed and permeabilized cells were stained with intracellular cytokine detection antibodies (IFN-γ [4S.B3], IL-2 [MQ1-17H12], and TNF-α [Mab11], all purchased from BD Biosciences, and Ki67 [Ki-67 from BioLegend) at RT for 20 min. Cell acquisition was performed on an LSRFortessa X20 or LSR II flow cytometer (BD Biosciences), and data were analyzed with Flowlogic software (Inivai, Mentone, Australia).

### IFN-γ ELISpot

ELISpot assays were performed using the human IFN-ELISpot^PLUS^ kit (Mabtech, Nacka Strand, Sweden), following the manufacturer’s instructions. Cultured cells were added (5 × 10^4^) to wells in duplicates and then stimulated with anti-CD3 mAb (positive control), irrelevant peptides (specificity control), test peptide, or vehicle only (DMSO). Spots were counted using an ELISpot reader (vSpot Reader Spectrum, AID, Straẞberg, Germany).

### Flow cytometry-based cytotoxicity assay

The flow cytometry-based cytotoxicity assay was performed using Cell Trace Violet (CTV)-labeled or Cell Trace Yellow (CTY)-labeled (Invitrogen) LMP2_426–434_ pulsed as target cells as described before.^45^ Briefly, target cells were prepared by stimulating autologous PBMCs with the T cell mitogen phytohemagglutinin (PHA) (3 × 10^6^ PBMCs/mL were incubated in T cell medium with 20 μg/mL PHA) for 3 days at 37°C and 5% CO_2_. Peptide-pulsed (or unpulsed) target cells were co-cultured with LMP2-specific T cells at various ratios for 4 h in CTL plus 10% horse serum. Unpulsed target cells alone were used as a control. After incubation, cells were harvested, stained using the LIVE/DEAD Fixable Aqua Dead Cell Stain Kit (Life Technologies) according to the manufacturer’s instructions, and viable target cells were quantified by flow cytometry using Flow Count Beads (Beckman Coulter, Brea, CA, USA). Cytotoxicity was calculated by comparing the percentage of viable target cells under test conditions relative to the control (100 − [target cell alive/target cell alone] × 100).

### scRNA-seq and high-throughput TCR sequencing

At the indicated time points, T cells were sorted based on MHC-Dextramer and CD8 staining using FACSAria III (BD Biosciences). The purity of the sorted population was 94%. A fraction of the sorted antigen-specific T cells was subjected to V(D)J and transcriptome scRNA-seq at the Genome Quebec facilities. Briefly, cells were counted, and viability was assessed using a hemocytometer and trypan blue. The targeted cell recovery was set at 6,000 cells, and libraries were prepared using the Chromium Next GEM Single Cell 5′ Kit v.2 and Chromium Single Cell Human TCR Amplification Kit (10X Genomics, Pleasanton, CA, USA) according to the manufacturer’s recommendations. Libraries were quantified using the Kapa Illumina GA with Revised Primers-SYBR Fast Universal Kit (Kapa Biosystems, Wilmington, MA, USA). The average size fragment was determined using a LabChip GX (PerkinElmer, Waltham, MA, USA) instrument. The 10X Single Cells 5′ libraries were sequenced on Illumina HiSeq 4000 PE28x98, and the 10X Single Cell V(D)J (Human T) libraries were sequenced on Illumina HiSeq 4000 PE150. The Illumina control software was HCS HD 3.4.0.38, and the real-time analysis program was RTA v.2.7.7. bcl2fastq2 v.2.20 was then used to demultiplex samples and generate fastq files. Reads were aligned to GRCh38 genome assembly, gene expression matrices were generated, and clonotype identification was performed using CellRanger v.3.0.2. The resulting gene expression matrices were normalized by total Unique molecular identifiers (UMI) counts per cell multiplied by the median UMI count per cell and natural-log-transformed using Scanpy v.1.4.^46^ PhenoGraph,^47^ clustering was applied with k = 20, and clusters with a median mitochondrial fraction greater than 0.2 were filtered out. Clonotypes were defined by nucleotide sequences of alpha and beta chains. To remove potential doublets, we restricted our analysis to cells in which exactly one alpha and one beta chain were identified. We retained only the most abundant clonotype for each alpha and beta chain. The number of retained cells per sample is provided in Table S2. After filtering, individual count matrices were combined, normalized, and log transformed, as described above, for downstream analyses. Pathway expression is defined as the log2 of the sum of the normalized gene expression of individual genes. Genes included with each pathway are listed in Table S3 (taken from Azizi et al.^17^). The significance of the changes in pathway expression between the control (CTRL) and DDB conditions was assessed using a Mann-Whitney U test. To set the threshold where p values are considered significant, we compared the p values obtained with the pathway of interest with those obtained with random sets of genes. For each pathway of interest, we generated 1,000 random gene sets with the same number of genes drawn from the same expression distribution. On each of these random gene sets, we performed the CTRL versus DDB comparison and recorded the p values obtained from the Mann-Whitney U test and report the percentile ranking of the p value in the pathway compared with the p values obtained from the random gene sets.

Bulk TCR repertoire profiling was performed from sorted multimer-positive and -negative CD8^+^ T cell RNA preserved in TRIzol™ reagent (Thermo Fisher Scientific) using next-generation sequencing (NGS) targeting the hypervariable CDR3 of TCRβ. TRIzol-extracted RNA, purified with the PureLink RNA Mini and Microcolumn System (Thermo Fisher Scientific), was quantified by UV spectrophotometry (Tecan), and tested for quality using with a Bioanalyzer chip (Agilent Technologies). TCRb amplicon libraries were prepared from 25 ng total RNA with the Oncomine TCR Beta-SR Assay for RNA (Thermo Fisher Scientific). The TCRb libraries obtained were quantified on the ViiA 7 real-time PCR system with the Ion Library TaqMan Quantitation Kit (Thermo Fisher Scientific). NGS was completed on the Ion S5 semiconductor platform using an Ion 540 chip prepared with the Ion Chef System (all from Thermo Fisher Scientific). TCRβ repertoire analysis was completed using the Ion Reporter software (Thermo Fisher Scientific) and Immunarch package (R software).^48^ The data for all genomic analyses can be found in the Gene Expression Omnibus (GEO: GSE182537 and GSE181682).

### Statistical analysis

Statistical significance was analyzed with the R software, v.4.0.4. Multiple-group comparisons were performed using one-way ANOVA and Tukey post hoc test or Kruskal-Wallis test with the Holm procedure to correct for multiple testing (when ANOVA requirements were not met). Unless stated otherwise, paired Wilcoxon-Mann-Whitney tests were performed for two-group comparisons. p values of 0.05 or less were considered significant.

## Data Availability

All genomic data are available in the Gene Expression Omnibus (GEO: GSE182537 and GSE181682), and the authors are willing to share the relevant data and codes used in this report.

## Supplemental information

Document S1. Figures S1–S12 and Table S1–S8

Table S3. Genes used for analysis

Document S2. Article plus supplemental information
