# Supplementary material for: Combined PD-L1 and TIM3 blockade improves expansion of fit human CD8+ antigen-specific T cells for adoptive immunotherapy
Source: Mol Ther Methods Clin Dev. 2022 Oct 4;27:230–45. doi: 10.1016/j.omtm.2022.09.016 (PMC9593254; doi:10.1016/j.omtm.2022.09.016)
Supplement: Document S1. Figures S1–S12 and Table S1–S8 [file mmc1.pdf]

OMTM, Volume *27*

Supplemental information

Combined PD-L1 and TIM3 blockade improves expansion of fit human CD8$^+$ antigen-specific T cells for adoptive immunotherapy

Shirin Lak, Valérie Janelle, Anissa Djedid, Gabrielle Boudreau, Ann Brasey, Véronique Lisi, Ali Smaani, Cédric Carli, Lambert Busque, Vincent-Philippe Lavallée, and Jean-Sébastien Delisle

Table S1: TCR alpha/beta chain sequences of the most abundant clonotypes across donors and conditions.

Clonotype ID	nt_TRA	nt_TRB	aa_TRA	aa_TRB	Donor
clonotype1	TGTGCTGCCCTCATGGATAGCAACTATCAGTTAATCTGG	TGCGCCAGCAGTGATGACGGGATGAACACTGAAGCTTTCTTT	CAALMDSNYQLIW	CASSDDGMNTEAFF	1 and 2
clonotype2	TGTGCTGTGCTCATGGATAGCAACTATCAGTTAATCTGG	TGCGCCAGCAGTGGGGACGGTATGAACACTGAAGCTTTCTTT	CAVLMDSNYQLIW	CASSGDGMNTEAFF	3
clonotype3	TGTGCAATGAGCGCGGAGGAGGACATGCGCTTT	TGTGCCAGCAGCTCCCGGCTAGCGGGGATCCAAACGAGCTCCTACAATGAGCAGTTCTTC	CAMSAEEDMRF	CASSSRLAGIQTSSYNEQFF	1 and 2
clonotype4	TGTGCAATGAGCGCGGAATCAAATTCCGGGTATGCACTCAACTTC	TGTGCCAGCAGCTTAGGGGGTTATGAGCAGTTCTTC	CAMSAESNSGYALNF	CASSLGGYEQFF	3
clonotype5	TGTGCAGGGGGCCGGGGCTGGGAGTTACCAACTCACTTTC	TGCGCCAGCAGCTTGGAGGGACAGGCGAGCTCCTACGAGCAGTACTTC	CAGAGAGSYQLTF	CASSLEGQASSYEQYF	2
clonotype6	TGTGCTGTGACTAACTTTGGAAATGAGAAATTAACCTTT	TGCGCCAGCAGCCAACTACAGGGATACGAGCAGTACTTC	CAVTNFGNEKLTF	CASSQLQGYEQYF	2
clonotype7	TGTGCAATGAGAGAGCCTCTCACGGGAGGAGGAAACAAACTCACCTTT	TGTGCCAGCAGTGGACTAGCGATCTCCTACGAGCAGTACTTC	CAMREPLTGGGNKLTF	CASSGLAISYEQYF	1

nt: nucleotide; aa: amino acid; TRA: TCR alpha chain; TRB: TCR beta chain.

Table S2: Single cell filtering results. Initial number of barcodes represents the output from CellRanger v3.0.2 while the number of cells after filtering corresponds to the cells kept for downstream analysis.

Sample	Initial number of barcodes	Number of cells after filtering
CTRL1	20182	12753
CTRL2	8550	1950
CTRL3	27019	9109
DB1	19724	10101
DB2	11107	2665
DB3	30813	8981

Table S3: Gene list used for analysis.

(Refer to appended spreadsheet)

Figure S1

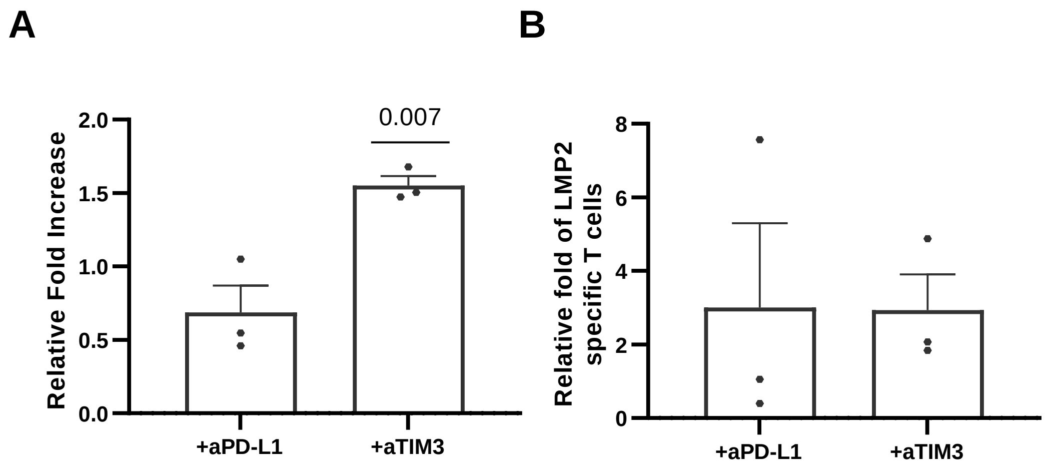

Figure S1. Delayed single TIM-3 blockade increases T-cell expansion but not antigen-specific T-cell yield. (A) Relative fold increase in cell counts at day 21 (control condition with no immune checkpoint blockade is set at 1) when anti-PD-L1 (aPDL1) or anti-TIM-3 (aTIM3) is introduced in the culture at day 7. (B) Similar analysis comparing antigen-specific T-cell yield. 3 different donors, significant p-values are indicated and reflect comparisons to the control condition (one-sample t-test), error bars indicate SEM

Figure S2

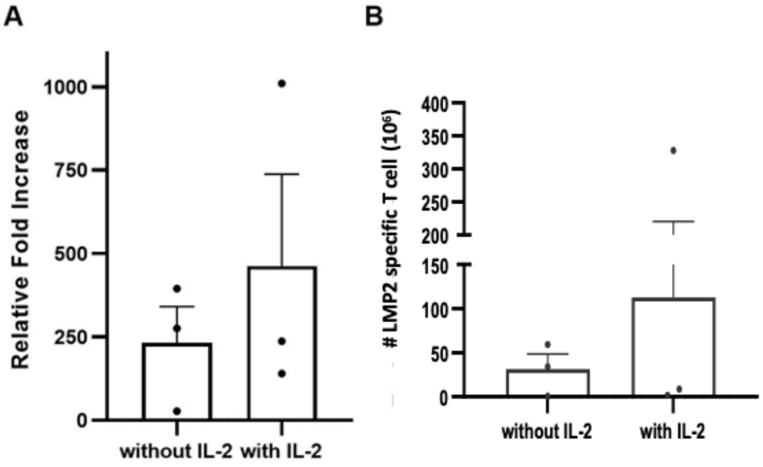

Figure S2. IL-2 does not consistently increase T-cell expansion in the DDB condition. (A) Relative fold increase calculated from input cell counts for the entire culture after 28 days of culture in the Delayed double blockade (DDB) condition with either no IL-2 supplementation (without IL-2) or IL-2 supplementation (with IL-2). (B) Absolute HLA-A0201-LMP2$_{426\text{-}434}$ multimer-positive T-cell yield at the end of the culture. No statistically significant differences were found. Error bars indicate SEM

Figure S3

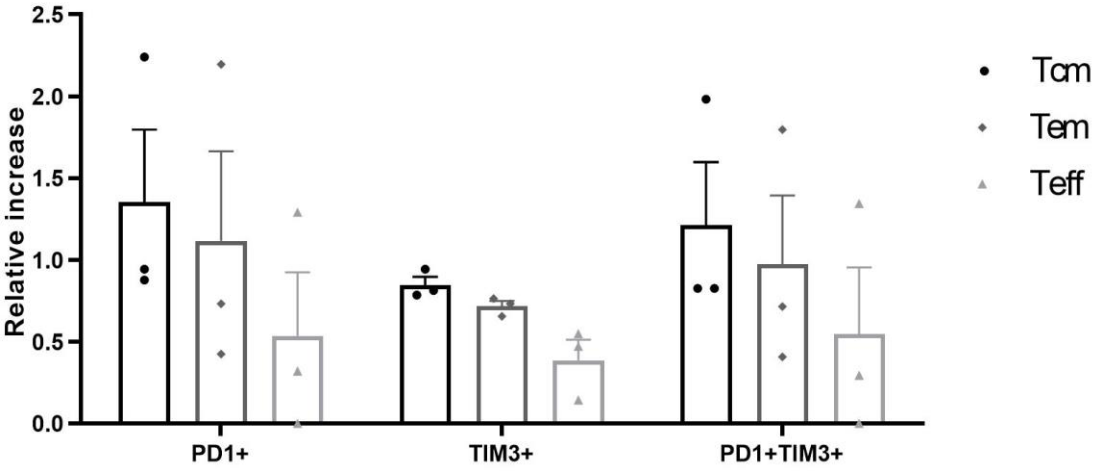

Figure S3. The expression of PD-1 and TIM-3 is similarly distributed among T-cell subsets, irrespective of experimental groups. Relative to the control condition (set at 1), fraction of HLA-A0201-LMP2$_{426-434}$ multimer positive T cells from the DDB condition expressing PD-1, TIM-3 or the combination of both for each subset (CD45RO+/CD62L+ central memory T cells – Tcm, CD45RO+/CD62L- effector memory T cells – Tem and CD45RO-/CD62L- effector T cells – Teff) No statistically significant differences were found. Error bars indicate SEM.

Figure S4

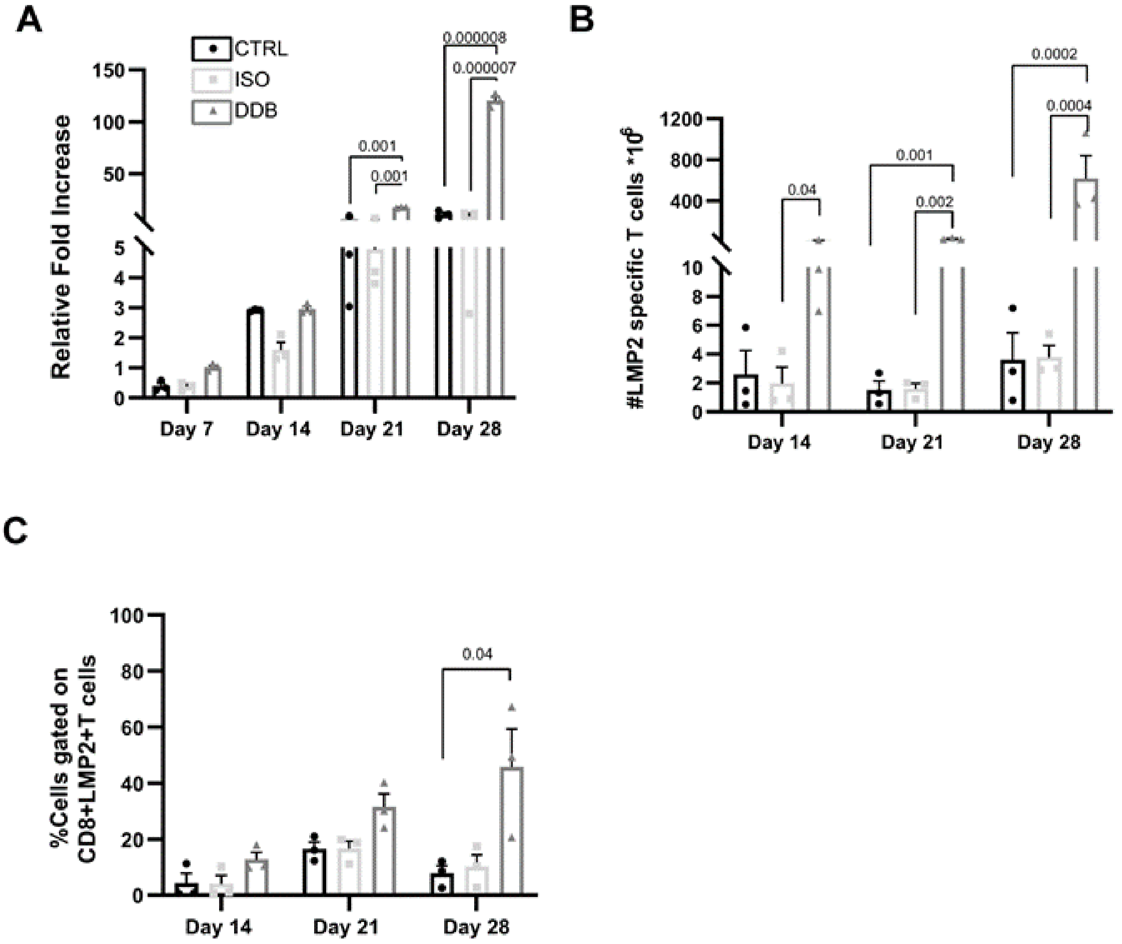

Figure S4. Validation of the DDB strategy using isotype control antibodies. (A) Cell expansion relative to input at the beginning of the culture ($15x10^{6}$) in function of time and culture condition; no antibodies (ctrl), isotypes (ISO) according to the delayed double blockade (DDB) schedule and with blocking antibodies (DDB). Absolute count (B) and percentage (C) of HLA-A0201-$LMP2_{426-434}$ (LMP2) multimer positive T cells in the same conditions and at the same time-points. 3 different donors, significant p-values are indicated, error bars indicate SEM.

Figure S5

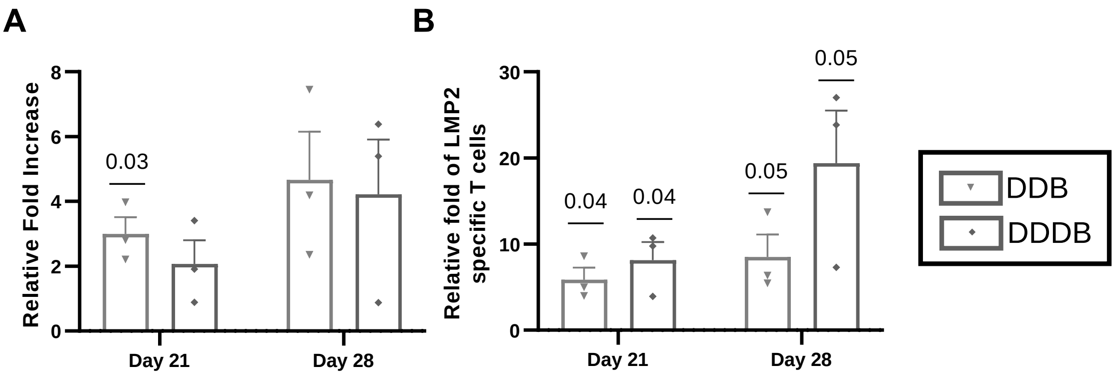

Figure S5. Double delayed double blockade (DDDB) is not superior to delayed double blockade (DDB). (A) Relative fold increase in cell counts at day 21 and 28 (standard condition with no immune checkpoint blockade set at 1) when anti-PD-L1 and anti-TIM-3 are introduced according to the delayed double blockade (DDB) scheme (anti-PD-L1 introduced at day 0 and anti-TIM-3 introduced at day 7) or when both antibodies are introduced at day 7 of the culture (DDDB). (B) Similar analysis comparing antigen-specific T-cell yield. 3 different donors, significant p-values are indicated and reflect comparisons to the control condition (one-sample t-test), error bars indicate SEM. No comparison between DDB and DDDB was found to be significant.

Figure S6

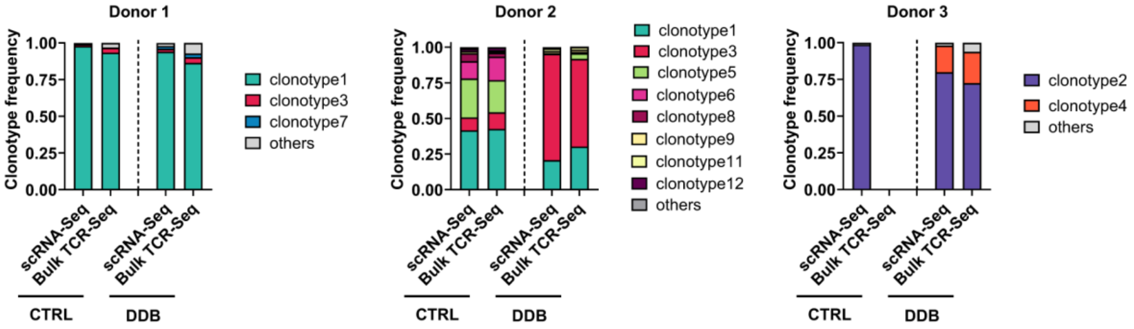

Figure S6. Correlation between clonotype determination using single-cell RNA sequencing (scRNA-seq) and bulk CDR3 beta-chain TCR sequencing. Comparison of clonotype repertoire among sorted HLA-A0201-LMP2$_{426-434}$ multimer positive T cells at day 28 from the control condition (CTRL) and delayed double blockade (DDB) condition according to scRNA-seq or bulk CDR3 sequencing (bulk TCR-Seq) for each donor. The bulk TCR-Seq from the CTRL condition in Donor 3 was not considered due to poor RNA quality.

Figure S7

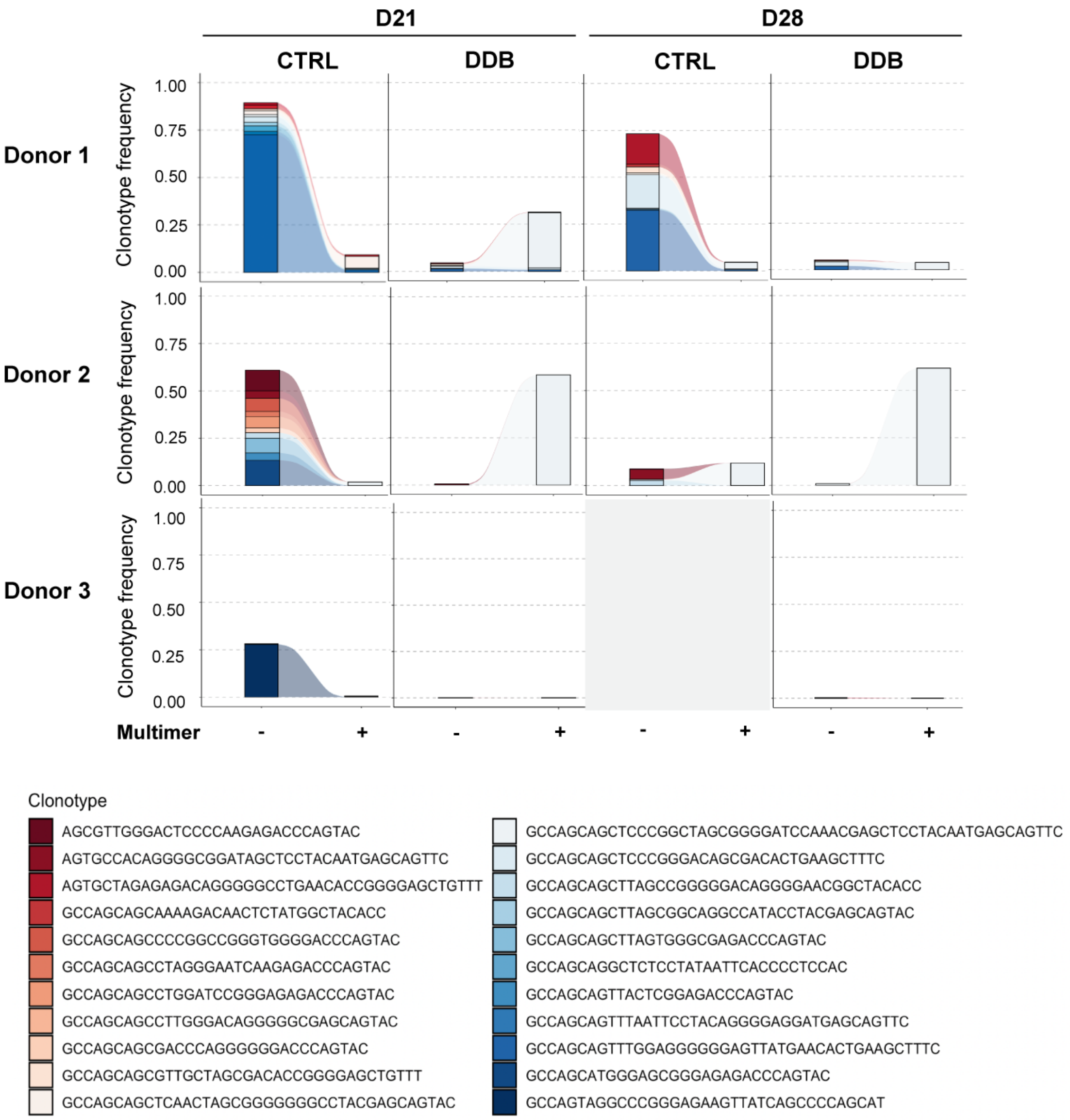

Figure S7. Limited overlap between HLA-A0201-LMP2$_{426-434}$ multimer positive and negative T cells. Bulk CDR3 beta-chain TCR sequencing data from sorted multimer positive (+) and negative (-) fractions from all donors in both experimental condition (control - CTRL and delayed double blockade – DDB) at the day 21 and 28 time points showing limited clonotype overlap. Clonotypes present in both fractions are listed and represented graphically. None of the clonotypes was present in both fractions simultaneously at high frequency (>10%). Due to poor RNA quality, the multimer-positive fraction of the control condition in Donor 3 could not be analyzed.

Figure S8

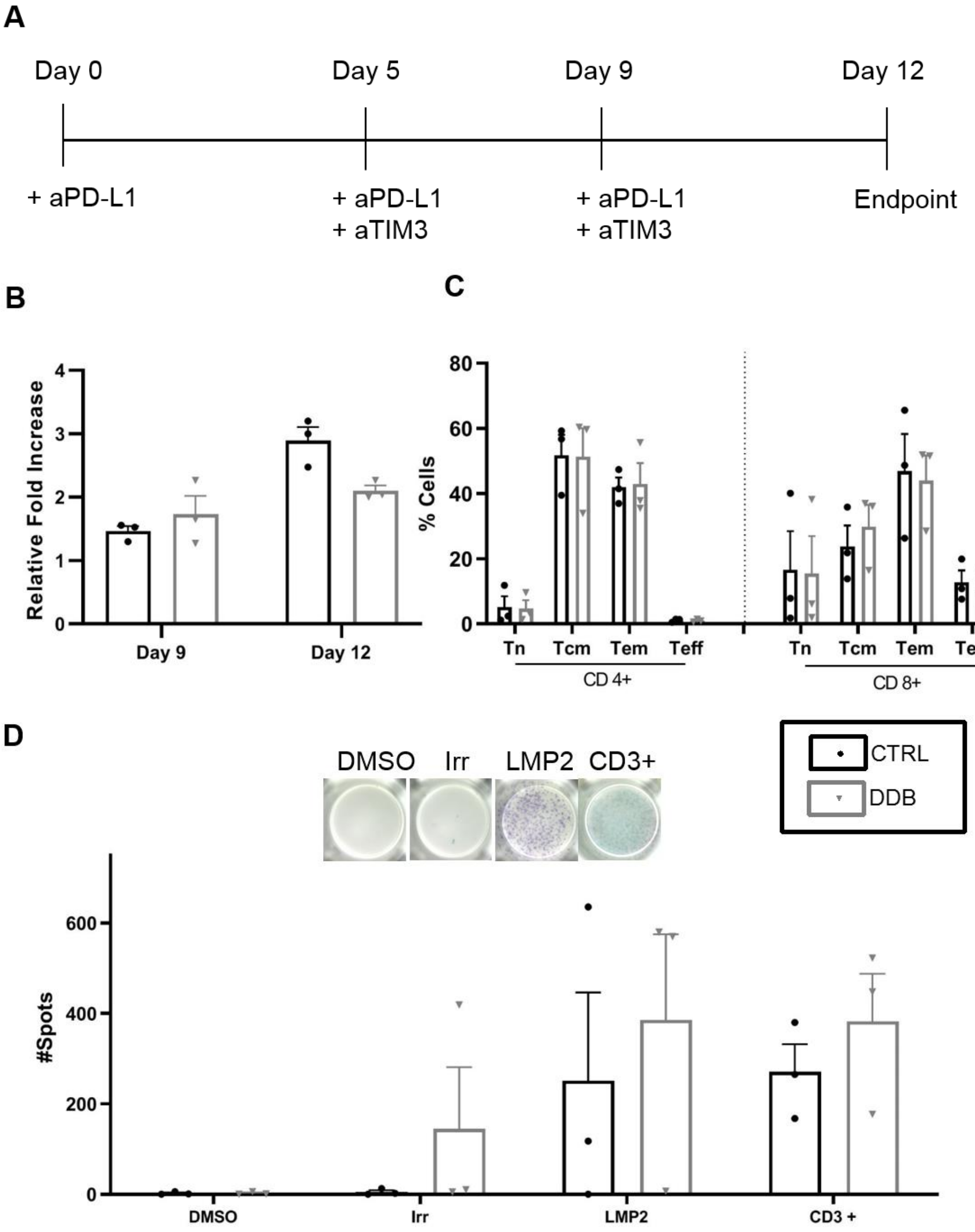

Figure S8. Double immune checkpoint blockade does not alter T-cell expansion, differentiation and reactivity of rapidly generated EBV-reactive T-cell lines. (A) Schematic representation of rapid T-cell line generation indicating media changes with the addition of antibodies. (B) T-cell expansion at day 9 and 12 relative to the input number of PBMC at day 0 in control (CTRL) cultures (no immune checkpoint blockade) set at 1 versus delayed double blockade (DDB) condition. (C) $CD4^{+}$ and $CD8^{+}$ T-cell differentiation according to CD62L and CD45RO expression (naïve; Tn, central memory; Tcm, effector memory; Tem and effector T cells; Teff). (D) ELISpot result using diluent (DMSO), a peptide library not used for T-cell stimulation, here cytomegalovirus pp65

peptide library (irrelevant, irr), the targeted library (LMP2) and positive control (agonistic anti-CD3 antibody – CD3). 3 independent donors, error bars represent SEM. No significant differences were found between CTRL and DDB.

Figure S9

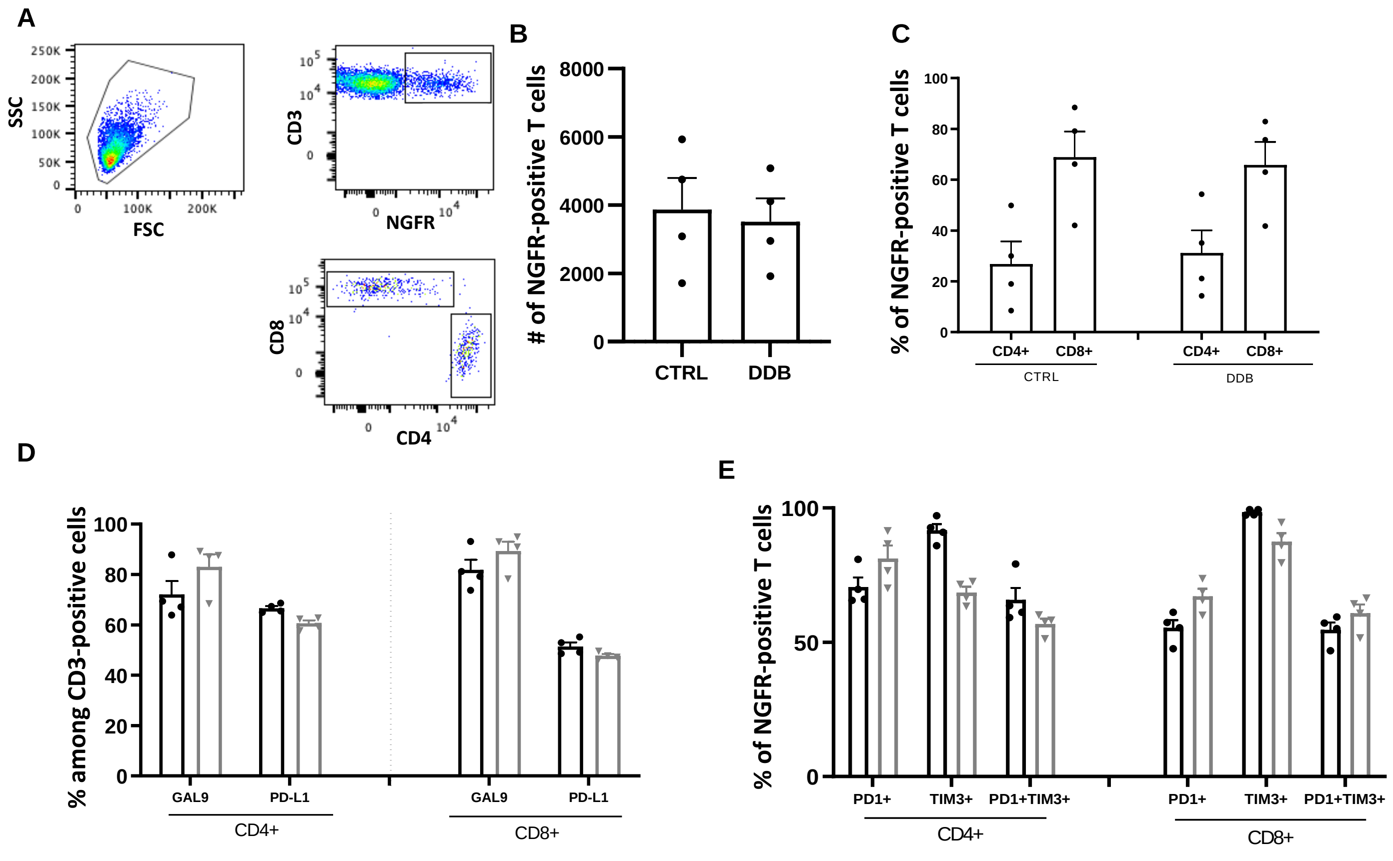

Figure S9. DDB in the context of CAR T cells production has no effect of T-cell expansion and phenotypes. (A) Representative results of CART cell gating (identified by non-signaling truncated Nerve growth factor receptor – NGFR reporter gene) positive T cells for CD4+ and CD8+ T-cell subset analysis. (B) Absolute counts of NGFR positive cells between conditions and (C) CD4+ and CD8+ subset distribution among CART cells. (D) Percentage expression among CD3-positive T cells in the culture. (E) Percentage expression of immune checkpoint on CART cells. 4 independent donors, error bars represent SEM. No significant differences were found between the control (CTRL) and Delayed double blockade condition (DDB).
